# Characterization of Changes and Driver Microbes in Gut Microbiota During Healthy Aging Using A Captive Monkey Model

**DOI:** 10.1016/j.gpb.2021.09.009

**Published:** 2021-12-30

**Authors:** Zhi-Yuan Wei, Jun-Hua Rao, Ming-Tian Tang, Guo-An Zhao, Qi-Chun Li, Li-Ming Wu, Shao-Qiang Liu, Bi-Hai Li, Bai-Quan Xiao, Xing-Yin Liu, Jian-Huan Chen

**Affiliations:** 1 Guangdong Key Laboratory of Animal Conservation and Resource Utilization, Guangdong Public Laboratory of Wild Animal Conservation and Utilization, Institute of Zoology, Guangdong Academy of Sciences, Guangzhou 510260, China; 2 Laboratory of Genomic and Precision Medicine, Wuxi School of Medicine, Jiangnan University, Wuxi 214122, China; 3 Joint Primate Research Center for Chronic Diseases, Jiangnan University and Institute of Zoology, Guangdong Academy of Sciences, Wuxi School of Medicine, Jiangnan University, Wuxi 214122, China; 4 Department of Pathogen-Microbiology Division, Nanjing Medical University, Nanjing 211166, China

**Keywords:** Age-dependent change, Non-human primate, Healthy gut microbiota, Network connectivity, Driver microbe

## Abstract

Recent population studies have significantly advanced our understanding of how age shapes the gut microbiota. However, the actual role of age could be inevitably confounded due to the complex and variable environmental factors in human populations. A well-controlled environment is thus necessary to reduce undesirable confounding effects, and recapitulate **age-dependent changes** in the gut microbiota of healthy primates. Herein we performed 16S rRNA gene sequencing, characterized the age-associated gut microbial profiles from infant to elderly crab-eating macaques reared in captivity, and systemically revealed the lifelong dynamic changes of the primate gut microbiota. While the most significant age-associated taxa were mainly found as commensals such as *Faecalibacterium*, the abundance of a group of suspicious pathogens such as *Helicobacter* was exclusively increased in infants, underlining their potential role in host development. Importantly, topology analysis indicated that the **network connectivity** of gut microbiota was even more age-dependent than taxonomic diversity, and its tremendous decline with age could probably be linked to healthy aging. Moreover, we identified key **driver microbes** responsible for such age-dependent network changes, which were further linked to altered metabolic functions of lipids, carbohydrates, and amino acids, as well as phenotypes in the microbial community. The current study thus demonstrates the lifelong age-dependent changes and their driver microbes in the primate gut microbiota, and provides new insights into their roles in the development and healthy aging of their hosts.

## Introduction

The human gut microbiota is composed of trillions of microbial cells that inhabit the gastrointestinal tract [1]. These microbes altogether encode an extremely large and dynamic genetic diversity, enabling the host to access additional energy and metabolites [2]. The gut microbiota thus plays a substantial role in human physiology and health [3]. In particular, commensal microbes in the gastrointestinal tract interplay with the host immune system, protect the host from pathogens, and modulate the host’s physiological functions with commensal-derived metabolites [4], [5], [6].

The development of human gut microbiota, with dynamic changes after birth, has been implicated to play an active role concomitantly with the host’s development and aging [7]. After first colonization at birth, the postnatal gut microbiota develops rapidly in the first few months of life [8], [9]. By one week of age, the infant gut microbiota has already become very similar to that at one month old [10]. Breastfeeding is one of the key factors that greatly shapes the infant gut microbiota, and is linked to the increase of *Bifidobacterium* species [11]. Analysis of fecal bacteria in human populations shows that changes may occur in the gut microbiota as age increases, which could be associated with increased risk of diseases, especially age-related diseases such as type II diabetes and hypertension in elderly people [7], [12], [13], [14].

Nevertheless, the actual effects of age on human gut microbiota remain to be further elucidated. The human gut microbial community is known to be extremely dynamic. Existing population-based studies are inevitably influenced by a number of confounding factors in the populations. The individual human microbiota pattern is vastly variable. And varying environmental factors, such as diets [15] and antibiotic use [16], could dramatically influence the bacterial community [17]. In addition, people of different generations in the same population may have distinct growth experiences and lifestyles due to the rapid urbanization of most human societies, which also shapes the human gut microbiota [18]. These confounding factors emphasize the difficulties and importance of studying healthy core native gut microbiota. A well-controlled model system that faithfully recapitulates age-dependent changes in the gut microbiota is thus needed, and would provide a better understanding of the roles played by the gut microbiota in the host’s healthy development and aging. In addition, humans have a much longer life span and dramatically different gut microbiota compared to rodents, the laboratory animals most widely used in existing gut microbiome studies [19]. In contrast, non-human primates (NHPs) are used as distinctive and indispensable model organisms in various areas of biomedical research and disease studies, given their high similarities to humans in terms of genetics, anatomy, reproduction, development, cognition, and social complexity [20]. For example, genome sequencing has shown that humans are 96% similar to the great ape species [21]. As for the gut microbiota, Li et al. reported that 39.49% of the non-redundant gut bacterial genes of crab-eating macaques (*Macaca fascicularis*) were found in the human gut bacterial genes. In contrast, only 0.6% of them were found in the mouse gut bacterial genes [22]. Such similarities in gut microbiota between humans and NHPs could be even increased by adopting human diets or living environments. Captivity allows the life and environment of animals to be more unified, and has been shown to humanize NHP gut microbiota [23]. Therefore, captive NHPs reared with a formula diet in a stable environment provide a feasible model to study age-dependent changes in the gut microbiota of humans and NHPs.

Various microbes in the gut microbiota interact to form a complex biological network. Therefore, not only taxonomic compositions, but also microbial interactions are essential to infer changes in microbial communities. In the current study, we conducted high-throughput sequencing of the 16S rRNA genes to analyze the fecal samples from captive infant, young-adult, middle-aged, and elderly crab-eating macaques, which are the widely used NHP animals sharing a high percentage of gut bacterial genes with humans [22]. Our results revealed the compositional, functional, and network topology changes of the gut microbiota associated with its maturation and development. Such findings could provide a baseline for a better understanding of gut microbiota changes in diseases.

## Results

### Age-dependent changes in gut microbiota diversity

To study the gut microbiota during healthy aging of crab-eating macaques, we used the 16S rRNA gene data to evaluate age-dependent changes of microbiota diversity, identify the top abundant and increased taxa as well as key driver microbes in different age groups, and explore age-associated microbial phenotypes and functions.

**Figure 1:**
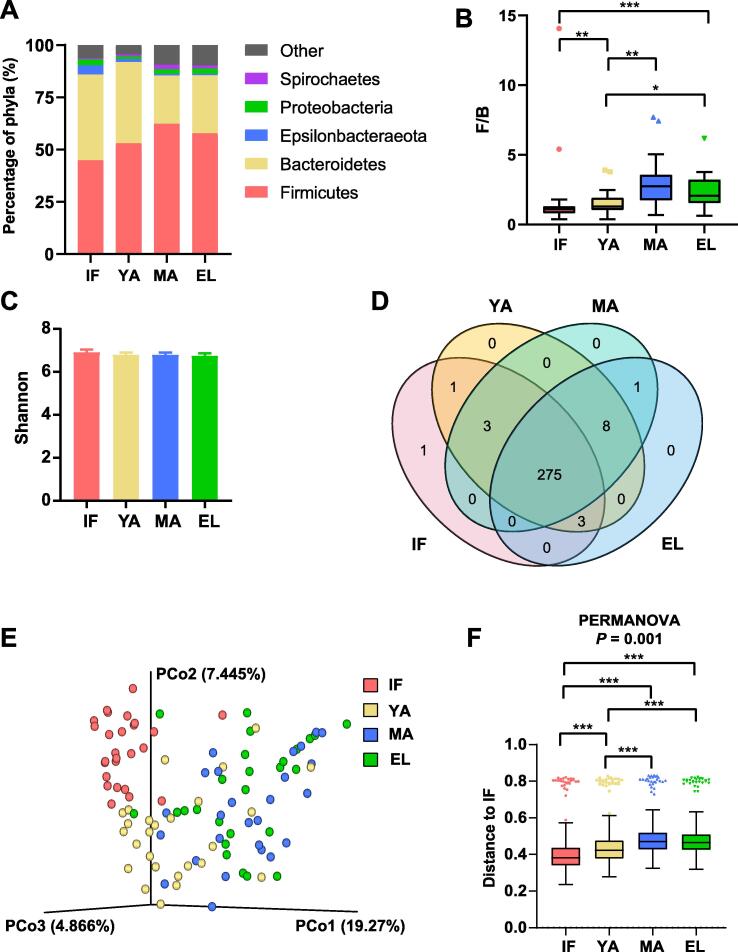
**Firmicutes**/**Bacteroidetes ratio andalpha/beta diversity in different age groups of macaques** **A.** Composition of gut microbiota at the phylum level in the four age groups. **B.** Bar chart showing the Firmicutes/Bacteroidetes (F/B) ratio for each age group. **C.** Shannon index in the four age groups. **D.** Venn plot illustrating the overlap of gut microbial genera among different age groups. Genera detected in more than six fecal samples are included. **E.** PCoA based on the Bray-Curtis distance matrix of all fecal samples. **F.** Unweighted Unifrac distance of gut microbiota between the three adult groups and the infant group. Pairwise *P* values are calculated using the nonparametric Kruskal-Wallis test with Tukey’s post-hoc test. IF, infant; YA, young-adult; MA, middle-aged; EL, elderly; PCoA, principal coordinate analysis; PERMANOVA, permutational multivariate analysis of variance. *, *P* < 0.05; **, *P* < 0.01; ***, *P* < 0.001.

The metadata of 16S rRNA gene sequencing of fecal DNA are summarized in Table S1. Rarefaction analysis of observed operational taxonomic units (OTUs) indicated that the sequencing efficiently captured the potential total OTUs in the fecal samples (Figure S1). The top five phyla observed in the fecal samples were Firmicutes (44.5%**–**61.1%), Bacteroidetes (26.4%**–**39.8%), Epsilonbacteraeota (2.3%**–**8.0%), Proteobacteria (1.9%**–**3.8%), and Spirochaetes (1.0%**–**2.7%) (Figure 1A), with Firmicutes and Bacteroidetes as the two dominant phyla. Furthermore, compared to infants, adults had a significantly increased Firmicutes/Bacteroidetes (F/B) ratio (all *P* < 0.05), especially in the middle-aged and elderly groups (infant, median = 1.09;  young-adult, median = 1.28; middle-aged, median = 2.74; elderly, median = 2.06; Figure 1B).

Comparison of metrics including Shannon index (Figure 1C, Figure S2), Pielou’s evenness, observed OTUs, phylogenetic diversity, and Simpson index (Figure S2) showed no significant change in alpha diversity among the four age groups. In line with this, the Venn diagram in Figure 1D showed that 275 (94.18%) genera detected in no less than ten fecal samples were shared across different ages (Table S2). As for beta diversity, infants mainly clustered separately from adults in the principal coordinate analysis (PCoA) based on the Bray-Curtis distance matrix, while the middle-aged and elderly groups clustered together with the young-adult samples falling in-between (Figure 1E). Furthermore, the permutational multivariate analysis of variance (PERMANOVA) results based on unweighted UniFrac distance indicated significant differences among the four age groups (Figure 1F). The inter-group unweighted UniFrac distance between adults and infants showed a trend similar to the F/B ratio (young-adult, median = 0.42; middle-aged, median = 0.47; elderly, median = 0.46), compared to the intra-group distance in infants (median = 0.38). These results thus pointed to remarkable microbial community changes associated with age.

### The top abundant genera in gut microbiota during aging

**Figure 2:**
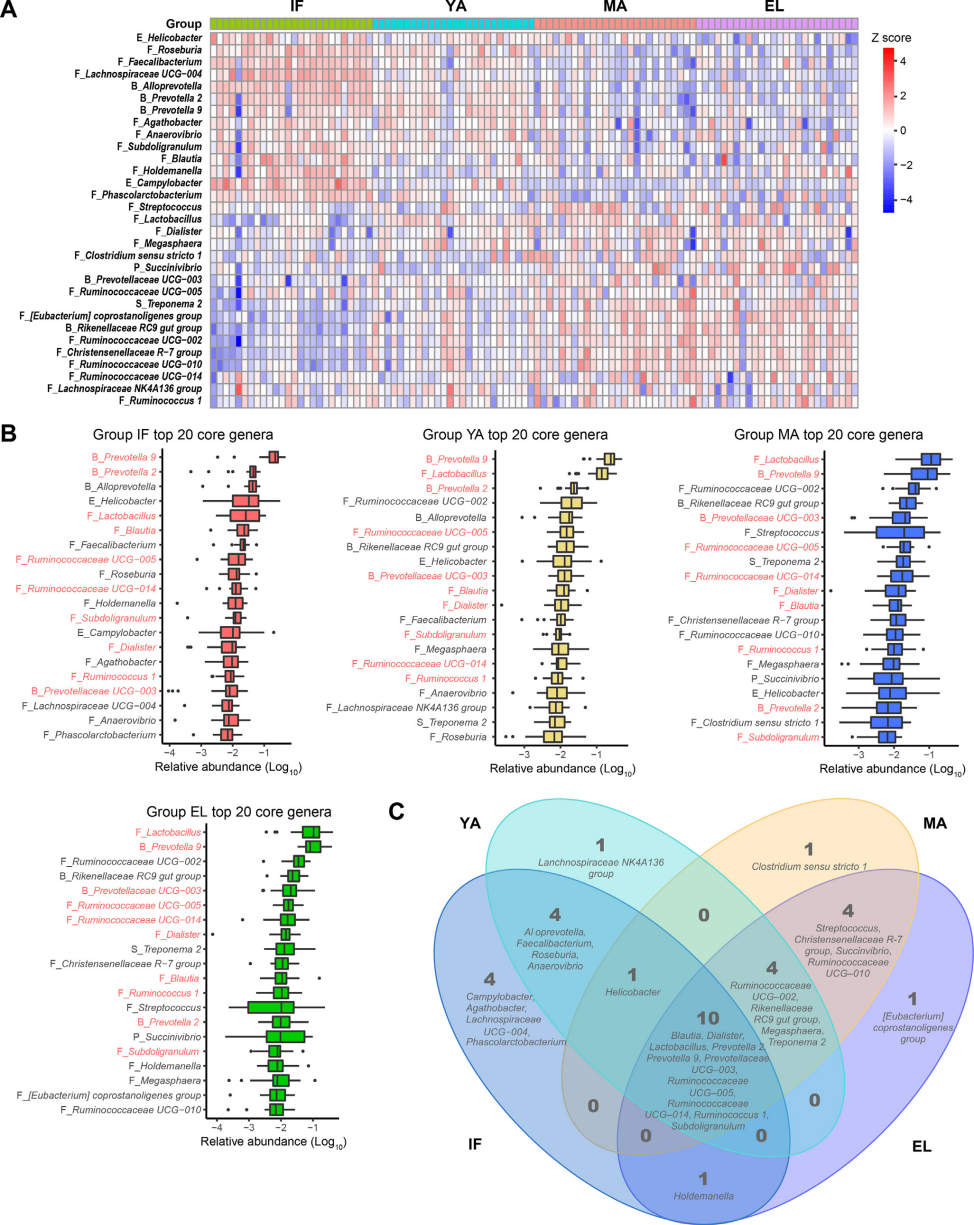
**The most abundant genera of gut microbiota indifferentage groupsA.** Heatmap showing the most abundant genera in the gut microbiota of the four age groups. **B.** Box plots showing the ranking of top 20 abundant genera in each of the four age groups. The ten genera shared by all four age groups are labeled in red. **C.** Venn plot illustrating the overlap of top 20 abundant genera among different age groups. The prefix before each genus name indicates the phylum which the genus belongs to. B, Bacteroidetes; F, Firmicutes; E, Epsilonbacteraeota; P, Proteobacteria; S, Spirochaetes.

We then focused on the most abundant genera during aging. Our results showed a trend of age-dependent changes in top abundant genera, similar to that of the beta diversity. The heatmap in Figure 2A showed the top 20 abundant genera from each of the four age groups, which were mainly commensals (Figure 2B). Half of these genera were shared by all age groups (Figure 2C), including four genera (*Ruminococcus 1*, *Ruminococcaceae UCG–005*, *Ruminococcaceae UCG–014*, and *Subdoligranulum*) from the Ruminococcaceae family, three genera (*Prevotella* *9*, *Prevotella* *2*, and *Prevotellaceae UCG–003*) from the Prevotellaceae family, *Lactobacillus*, *Blautia*, and *Dialister*.

We also looked into *Bacteroides*, which has been reported to be abundant in the gut microbiota of humans living in developed countries [24]. However, this genus showed a low median abundance of less than 0.1% in our captive macaques (data not shown).

### Correlation between differentially abundant gut microbes and age

**Figure 3:**
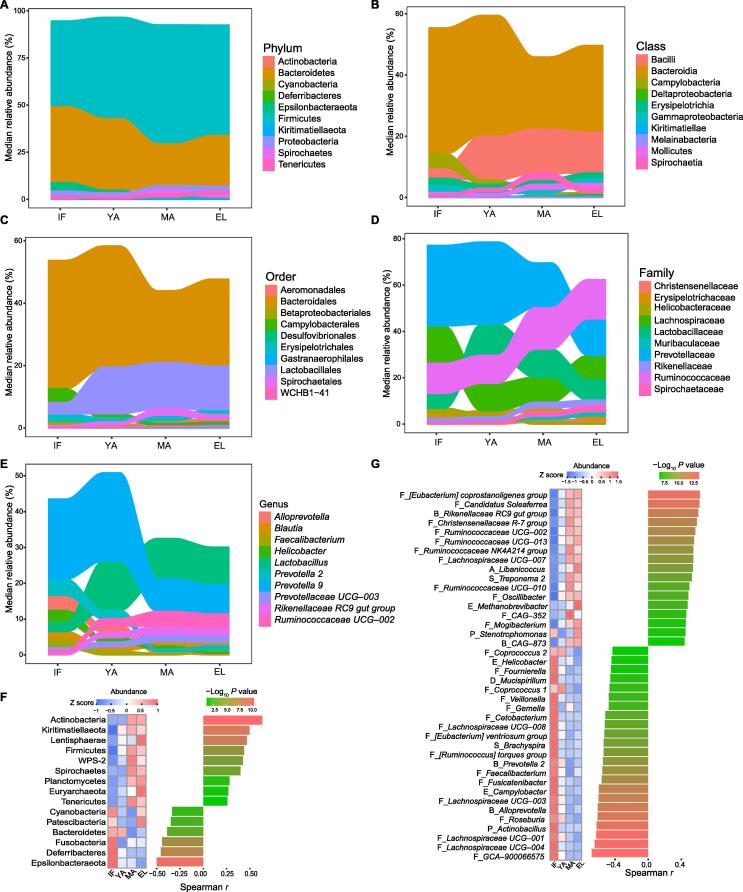
**Correlation between differentially abundant gut microbes and age** **A.**–**E.** Alluvial plots illustrating age-dependent phylogenetic shifts of the top 10 differentially abundant taxa at the phylum (A), class (B), order (C), family (D), and genus (E) levels. Differentially abundant taxa are ranked by their median of abundance. **F.** and **G.** Heatmaps showing significant age correlations for differentially abundant phyla (F) and genera (G) with FDR < 0.05. *P* values are calculated by the Spearman correlation test. For genera, only the top 40 genera ranked by |*r*| are shown. FDR, false discovery rate.

To further characterize the dynamic gut microbiota changes during aging, we analyzed the correlation of OTUs with age as a continuous variable. OTUs with significantly different abundance among age groups were firstly identified using STAMP (Figures S3 and S4). The alluvial plots in Figure 3A–E illustrated age-dependent shifts of these taxa at different taxonomic levels. The correlation of their abundances with age was then analyzed using Spearman correlation. At the phylum level (Figure 3F, Figure S5), Epsilonbacteraeota, Deferribacteres, Fusobacteria, Bacteroidetes, Patescibacteria, and Cyanobacteria were negatively associated with age, while Actinobacteria, Kiritimatiellaeota, Lentisphaerae, Firmicutes, WPS–2, Spirochaetes, Planctomycetes, Euryarchaeota, and Tenericutes were positively associated with age. At the genus level, in total, 112 genera were significantly associated with age, among which 29 were from the Lachnospiraceae family, and 18 were from the Ruminococcaceae family (Figure S6). A large proportion of the genera negatively associated with age were from the Lachnospiraceae family. The top 40 genera with the strongest correlations with age were shown in Figure 3G. Among these microbes, 23 genera were negatively associated with age, most of which were potential commensals. These microbes included 11 genera from the Lachnospiraceae family (*Lachnospiraceae UCG–001*, *Lachnospiraceae UCG–003*, *Lachnospiraceae UCG–004*, *Lachnospiraceae UCG–008*, *[Eubacterium] ventriosum group*, *Fusicatenibacter*, *GCA–900066575*, *[Ruminococcus] torques group*, *Coprococcus 1*, *Coprococcus 2*, and *Roseburia*), two genera from the Prevotellaceae familly (*Alloprevotella* and *Prevotella 2*), two genera from the Ruminococcaceae family (*Faecalibacterium* and *Fournierella*), *Actinobacillus*, *Campylobacter*, *Helicobacter*, *Mucispirillum*, *Veillonella, Cetobacterium*, *Brachyspira*, and *Gemella*. These top age-associated genera also included 17 genera positively associated with age, including six from the Ruminococcaceae family (*Ruminococcaceae UCG–002*, *Ruminococcaceae UCG–010*, *Ruminococcaceae UCG–013*, *Ruminococcaceae NK4A214 group*, *CAG–352*, and *[Candidatus] Soleaferrea group*), *CAG–873*, *Treponema 2*, *Methanobrevibacter*, *Rikenellaceae RC9 gut group*, *Christensenellaceae R-7 group*, *[Eubacterium] coprostanoligenes group*, *Lachnospiraceae UCG–007*, *Libanicoccus*, *Oscillibacter*, *Mogibacterium*, and *Stenotrophomonas*.

In addition, we also found a significant correlation between age and the abundance of lactic acid-producing bacteria, a group of probiotics in humans (Figure S6). Both *Bifidobacterium* and *Lactobacillus* increased with age (*r* = 0.34, *P* = 4.2 × 10^−4^ and *r* = 0.29, *P* = 0.0025, respectively).

### Differential taxa of gut microbiota in the four age groups

**Figure 4:**
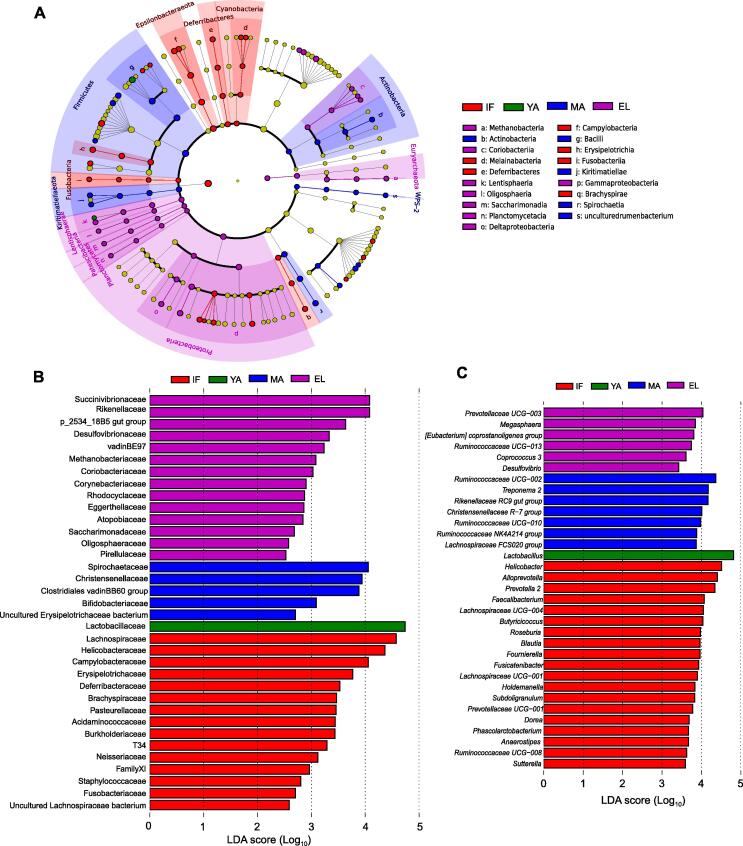
**Differentially abundant taxa increased in the four age groups** **A.** Phylogenetic cladogram showing differentially abundant taxa from kingdom to family levels. Microbial classes are indicated with letters. **B.** and **C.** Bar charts showing differentially abundant taxa in four age groups at the family (B) and genus (C) levels with average abundance > 0.1%. LDA, linear discriminant analysis.

We then utilized linear discriminant analysis effect size (LEfSe) to identify differential taxa that showed the highest abundance in each of the four age groups. At the phylum level, Epsilonbacteraeota and Cyanobacteria showed the highest abundance in the infant group; Firmicutes, Actinobacteria, and Kiritimatiellaeota were the most abundant in the middle-aged group; and Proteobacteria and Euryarchaeota showed the highest abundance in the elderly group (Figure 4A). No phylum showed a significantly higher abundance in the young-adult group.

Infant macaques showed the largest numbers of differentially abundant families and genera (average abundance > 0.1%) across the four age groups (Figure 4B and C). Nineteen genera showed the highest abundance in the infant group: seven genera from the Lachnospiraceae family (*Anaerostipes*, *Blautia*, *Dorea*, *Fusicatenibacter*, *Lachnospiraceae UCG–001*, *Lachnospiraceae UCG–004*, and *Roseburia*), five genera from the Ruminococcaceae family (*Butyricicoccus*, *Faecalibacterium*, *Fournierella*, *Ruminococcaceae UCG–008*, and *Subdoligranulum*), three genera from the Prevotellaceae family (*Alloprevotella*, *Prevotella 2*, and *Prevotellaceae UCG–001*), *Helicobacter* from the Helicobacteraceae family, *Holdemanella* from the Erysipelotrichaceae family, *Phascolarctobacterium* from the Acidaminococcaceae family, and *Sutterella* from the Burkholderiaceae family. As for the young-adult group, family Lactobacillaceae and genus *Lactobacillus* showed the highest abundance. It was noticed that Bifidobacteriaceae, another group of important lactic acid-producing bacteria, and four other families showed the highest abundance in the middle-aged group. Seven genera showed the highest abundance in the middle-aged group, including three from the Ruminococcaceae family (*Ruminococcaceae NK4A214 group*, *Ruminococcaceae UCG–002*, and *Ruminococcaceae UCG–010*), *Treponema 2* from the Spirochaetaceae family, *Rikenellaceae RC9 gut group*, *Christensenellaceae R-7 group*, and *Lachnospiraceae FCS020 group*. In the elderly group, the most abundant family was Succinivibrionaceae, and six genera showed the highest abundance, including *Prevotellaceae UCG–003*, *Ruminococcaceae UCG–013*, *Megasphaera* from the Veillonellaceae family, *Coprococcus 3* from the Lachnospiraceae family, *Desulfovibrio* from the Desulfovibrionaceae family, and *[Eubacterium] coprostanoligenes group*.

### Age-dependent gut microbiota networks and their key driver genera

**Figure 5:**
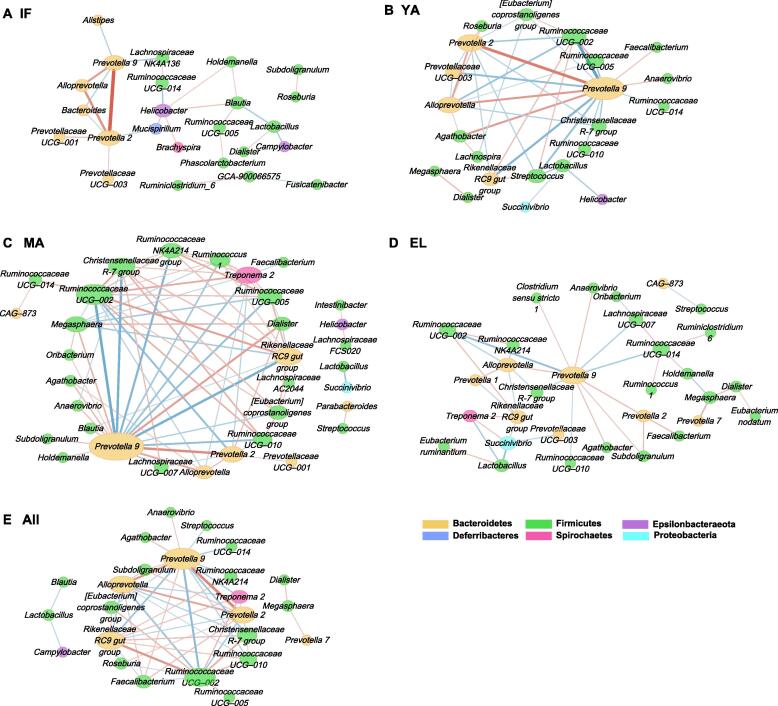
**The interactive networks of gut microbiota** The interactive networks of gut microbiota in the infant (**A**), young-adult (**B**), middle-aged (**C**), and elderly (**D**) groups, as well as in all fecal samples (**E**), are constructed from the SparCC results, and visualized using Cytoscape. Genera with average abundance > 0.1%, correlation |*r*| > 0.2, and *P* < 0.05 are included in the networks. Node color denotes the phylum of a certain genus. Node size represents the weighted node connectivity. Edge color and thickness represent correlation *r*. SparCC, sparse compositional correlation.

**Figure 6:**
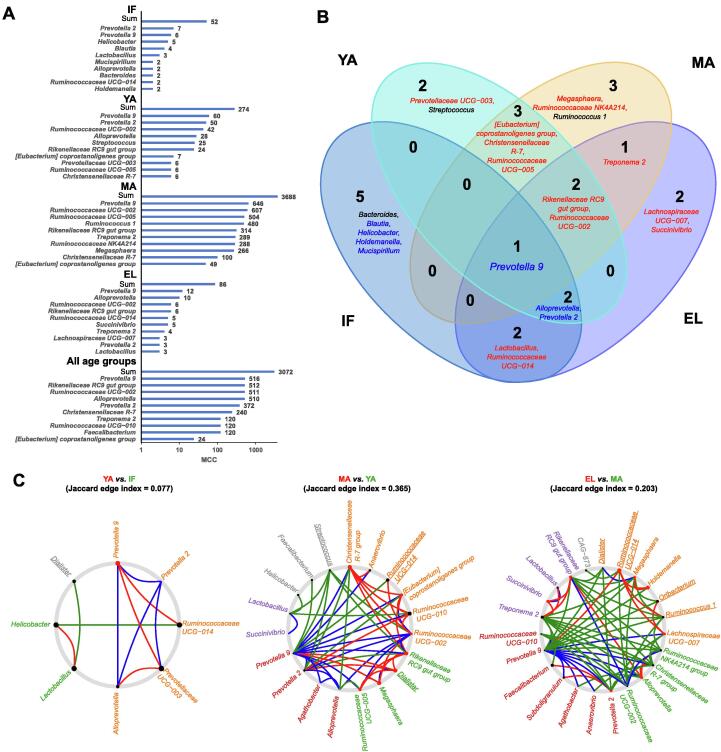
**Topological analysis identifies hub and driver genera in microbiota networks** **A.** MCC scores from the whole network and top 10 hub genera in the SparCC networks for the four age groups and all samples. **B.** Venn plot showing the overlap of hub genera among the four age groups. Genera are colored blue if negatively associated with age, and red if positively associated with age. **C.** NetShift common sub-networks based on the SparCC networks with highlighted driver genera. Node sizes are in proportion to their NESH scores, and the nodes representing the potential drivers are highlighted in red. Edges are colored in red if present only in case, in green if present only in control, and in blue if present in both. Node names without underlines denote age-associated genera. MCC, maximal clique centrality; NESH, neighbor shift.

We then further performed the sparse compositional correlation (SparCC) analysis and weighted correlation network analysis (WGCNA) to explore the interactions among gut microbes in the four age groups (Figure 5, Figure S7). All genera with relative abundance > 0.1% were included in the networks. Surprisingly, although not preferentially selected, the age-associated genera were found to be the major components of these networks. The gut microbiota network in the infant group had the lowest connectivity, as indicated by its small maximal clique centrality (MCC) score (total MCC score = 52) (Figure 5A, Figure 6A). The network developed into a more mature stage in the young-adult group (total MCC score = 274) (Figure 5B, Figure 6A), and had the highest connectivity in the middle-aged group (total MCC score = 3688) (Figure 5C, Figure 6A). Unexpectedly, although similar gut microbiota diversities were found between the elderly and middle-aged groups, the network connectivity dramatically decreased in the elderly group (total MCC score = 86) (Figure 5D, Figure 6A). The WGCNA results identified clusters (modules) in the gut microbiota of different age groups (Figure S7; Table S3).

We then utilized cytoHubba to analyze the hub genera, which were supposed to be identified by ranking their MCC and EcCentricity (EC) scores. Among the hub genera shown in Figure 6A, *Prevotella 9* was the only one shared by all four age groups as well as the network constructed using all samples (Figure 6A and B). The inter-genera interactions mediated by *Prevotella 9* could be of potential importance. The strongest positive interactions in the microbial communities were found between *Prevotella 2* and *Prevotella 9* and between *Alloprevotella* and *Prevotella 9* in the infant group. In addition to *Prevotella 9*, *Helicobacter* and *Prevotella 2* were another two important hub genera in the infant group. The role of such interactions mediated by these genera, particularly *Prevotella 9*, gradually diminished with age, and were in part replaced by interactions mediated by the hub genera negatively associated with age, such as *Ruminococcaceae UCG–002* and *Rikenellaceae RC9 gut group*.

Moreover, we performed the NetShift analysis to detect rewiring among the microbiota SparCC networks, and identified key driver microbes responsible for the changes (Figure 6C; Table S4). *Prevotella 9* was found to be the only driver genus responsible for the microbial changes between the infant and young-adult groups. Novel interactions with *Prevotella 9* were established in the gut microbiota of the young-adult group compared to that of the infant group. As for adults, multiple potential drivers were identified. Among these drivers, *Rikenellaceae RC9 gut group* and *Megasphaera* were the two key driver genera that contributed to the long-term development of gut microbiota in adults. Another five genera, including *Dialister*, *Christensenellaceae R-7 group*, *[Eubacterium] coprostanoligenes group*, *Ruminococcaceae UCG–005*, and *Ruminococcaceae UCG–002 group*, were involved in the change of gut microbiota between the young-adult and middle-aged groups. Another six genera, including *Ruminococcaceae UCG–014*, *Holdemanella*, *Succinivibrio*, *Alloprevotella*, *Lachnospiraceae UCG–007*, and *Prevotella 2*, were involved in the change of gut microbiota between the middle-aged and elderly groups.

### Age-associated phenotypes and functions of gut microbiota

**Figure 7:**
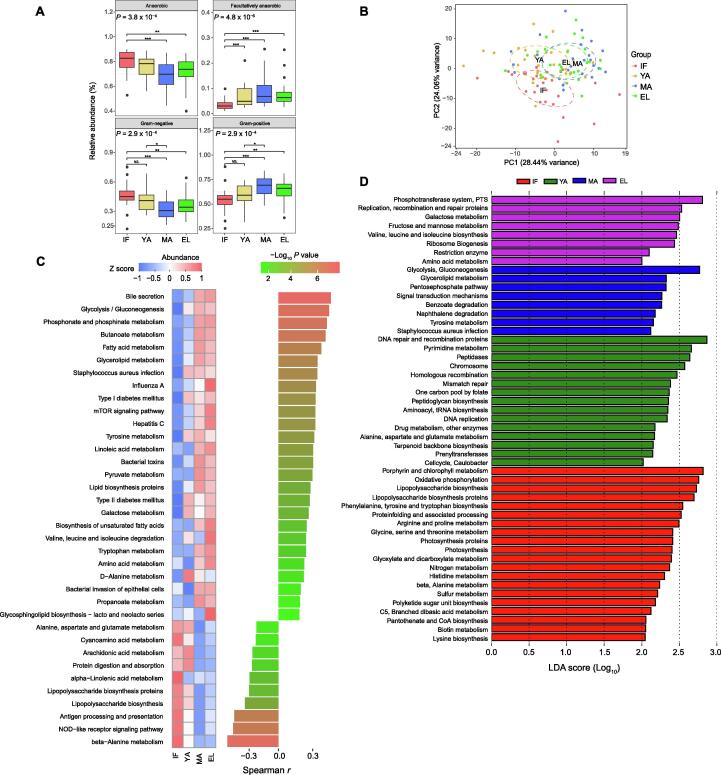
**Age-associated gut microbial phenotypes and functional profiles** **A.** Comparison of gut microbes with different phenotypes predicted by BugBase among the four age groups. *P* values for group comparisons are calculated by the nonparametric Kruskal-Wallis test with Tukey’s post-hoc test. *, *P* < 0.05; **, *P* < 0.01; ***, *P* < 0.001; NS., not significant. **B.** PCA plot based on microbial function profiles predicted by the PICRUSt software. **C.** Heatmap illustrating the median abundance and age correlation of gut microbial functions related to the metabolism of carbohydrates, lipids, and proteins as well as host immune response. *P* values are calculated by the Spearman correlation test. Pathways with FDR < 0.05 are shown. **D.** LEfSe results of gut microbial functions enhanced in each of the four age groups. PCA, principal component analysis; PICRUSt, Phylogenetic Investigation of Communities by Reconstruction of Unobserved States.

To understand the potential functional impact of age-dependent taxonomic changes in the gut microbiota, the microbial phenotypes were predicted using BugBase and compared among different age groups. The relative abundances of microbes with the anaerobic and Gram-negative phenotypes were significantly down-regulated, whereas the relative abundences of microbes with the facultative anaerobic and Gram-positive phenotypes were up-regulated in the middle-aged and elderly groups compared to the infant group (all *P* < 0.01) (Figure 7A). In line with these findings, Spearman correlation analysis showed that, the relative abundances of microbes with the anaerobic and Gram-negative phenotypes significantly decreased with age (*r* = −0.37, *P* = 1.2 × 10^−4^ for anaerobic; *r* = −0.34, *P* = 4.3 × 10^−4^ for Gram-negative), whereas the relative abundances of microbes with the facultative anaerobic and Gram-positive phenotypes significantly increased with age (*r* = 0.42, *P* = 8.7 × 10^−6^ for facultative anaerobic; *r* = 0.34, *P* = 4.3 × 10^−4^ for Gram-positive) (Figure S8).

We also determined the age-dependent changes in gut microbial functions using the Phylogenetic Investigation of Communities by Reconstruction of Unobserved States (PICRUSt) software, and identified 152 Kyoto Encyclopedia of Genes and Genomes (KEGG) modules to be significantly associated with age (Table S5). The principal component analysis (PCA) plot derived from the abundance of KEGG modules revealed remarkable differences in microbial functions among different age groups, showing a similar pattern with beta diversity (Figure 7B). We observed a significant correlation between these microbial functions and age. As shown in the heatmap in Figure 7C, metabolic pathways that were most positively associated with age were mainly involved in the biosynthesis and metabolism of lipids, carbohydrates, and amino acids. And metabolic pathways that were most negatively associated with age were mainly involved in host immune response and biosynthesis of the immunomodulating metabolite lipopolysaccharides (LPSs), which are endotoxin derived from the outer membrane of Gram-negative bacteria. LEfSe analysis further showed that the pathways related to porphyrin and chlorophyll metabolism, oxidative phosphorylation, and biosynthesis of LPSs were up-regulated in the infant group (Figure 7D). In contrast, biosynthesis of peptidoglycan, another important immunomodulating metabolite mainly derived from Gram-positive bacteria, was increased in the young-adult group. Metabolism of carbohydrates was most up-regulated in the middle-aged and elderly groups. Noteworthy, strong correlations were found between these age-associated microbial functions and gut microbes, particularly for the hub and driver genera (Figure S9), with the largest number of positive correlations found in *Prevotella 9*.

## Discussion

By using the NHP model of captive crab-eating macaques, we revealed remarkable lifelong age-dependent changes in gut microbial compositions and functions. Moreover, our study identified hub and driver microbes that hold a potential significance in the age-dependent microbial interplay. Given the similarities between captive crab-eating macaques and humans, these findings could provide a better understanding of age-dependent changes in the human gut microbiota.

The gut microbiota of captive macaques in this study showed similarities to that of humans, especially those in developing countries [12], [23], [25], [26]. In line with those of humans and other NHPs [1], [27], the gut microbiota of our captive crab-eating macaques was dominated by Firmicutes and Bacteroidetes across all ages (Figure 1A). Most of the common genera with high abundances across all ages are potentially commensals from the Ruminococcaceae and Prevotellaceae families (Figure 2B). In contrast, the abundance of *Bacteroides* was very low. Gut microbial communities of individuals from developing countries were reported to be dominated by *Prevotella* [26], while those from developed countries were highly abundant in *Bacteroides* [12]. Plant-based diets with low fat could be involved in the higher similarities between the gut microbiota of captive macaques and humans living in developing countries [24].

The lack of significant changes in alpha diversity might indicate the importance of microbiota studies in captive NHPs (Figure 1C). Yatsunenko et al. [12] reported that observed OTUs increased with age in the gut microbiota of all three populations. In a recent gut microbiota study of non-captive rhesus monkeys, Chen et al. [28] reported that male adults had significantly higher Shannon index than male juveniles. However, under a well-controlled environment provided by captivity, alpha diversity changes are probably smoothed out. By the age of 1–2 years old, infant gut microbiota had gained more than 94% of genera observed in adults (Figure 1D). Age-related factors, such as diets and lifestyles, rather than age itself, might actually contribute to the age-associated increase of alpha diversity in human populations.

Nevertheless, the remarkable age-dependent changes, including the F/B ratio and beta diversity as well as network topology, emphasized the actual effects of age on the gut microbiota in captive macaques (Figure 1B). The F/B ratio is considered as an indicator of the maturation and development of gut microbiota [29], and has been reported to be involved in health-related conditions or diseases such as obesity [30]. In the current study, the F/B ratio increased in adult macaques, and decreased in elderly macaques, resembling the observation in humans [29], [31]. It could be due to increased Firmicutes and decreased Bacteroidetes with age (Figure 3F). Interestingly, although middle-aged and elderly macaques had similar beta diversity, evident reduction of connectivity in elderly macaques indicated a decline of microbial interactions. Such findings suggest that network connectivity could be more sensitive than the F/B ratio and biological diversity to detect age-dependent changes in the gut microbiota.

Moreover, the age-associated microbes identified in captive macaques could be involved in the host’s development and aging in good health (Figures 3 and 4). These microbes could play distinct roles depending on their direction of age correlation. A large proportion of these age-associated genera decreased with age, including those increased in infants. The compositions and activities in the infant gut microbiota have been engaged in the host’s early development and a variety of diseases, such as allergy and autism [5], [32], [33]. These genera negatively associated with age in fact consisted of at least two distinct groups. First, these genera contained potential commensals, which were active players in the early development of gut microbiota. The interplay between these commensals and the host intestinal barriers is important for the postnatal development of host metabolism, immunity, and mucosal barrier [34], [35], [36]. Commensals could benefit the host by producing metabolites such as short-chain fatty acids [37]. A number of the age-associated commensals in the current study are butyrate-producing bacteria in the host colon, including *Faecalibacterium*, *Roseburia*, *Anaerostipes*, and *Butyricicoccus* [38]. These commensals include anti-inflammatory bacteria, and outcompete pathogens to protect the host, and abnormal alteration of them have been reported in various human diseases [39], [40], [41], [42], [43]. For example, *Faecalibacterium prausnitzii*, one of the most abundant anti-inflammatory commensal bacteria in the colon, was reduced in Crohn’s disease patients [41]. Second, these bacteria negatively associated with age also contained a number of suspicious pathogens, especially enteropathogens. *Campylobacter* and *Actinobacillus* are causes of infectious diseases in humans [44]. Species from the *Brachyspira* genus are known pathogens causing diarrhea in animals and humans [45]. Bacteria from the *Gemella* genus are involved in endocarditis [46]. *Anaerobiospirillum succiniciproducens* from the *Anaerobiospirillum* genus has been found to be associated with diarrhea and bacteremia [47]. In this study, *Helicobacter*, a group of Gram-negative bacteria, was identified as a hub genus with a high abundance in infant gut microbiota, but its role remained largely unclear. *Helicobacter macacae* from this genus has been reported to be frequently detected in rhesus monkeys without a diarrheal history [48]. Rhoades et al. [9] reported that *Helicobacter macacae* was increased in 8-month infants that remained asymptomatic for diarrhea. In line with these findings, the biosynthesis of LPSs was also up-regulated in our infant macaques (Figure 7), further supporting a potential role of these age-associated microbes in the modulation of the host’s immunity. It should be taken into account that all macaques in the current study were in good health. Therefore, the gradual decrease of these suspicious pathogens with age might be associated with the maturation of the gut mucosal barrier. In addition, recent studies have reported possible effects of pathogens protecting the host against allergic sensitization [49], [50]. In our captive macaques, the suspicious pathogens with their abundances under control might allow “good” exposure for the proper training of the host’s immune system.

In the current study, while the roles of the microbes positively associated with age remained largely unclear, they could be related to the host’s healthy aging. A subset of these microbes has been implicated in the metabolism of nutrients, including lipids and carbohydrates, which is in line with the predicted gut microbial functions up-regulated with age in our macaques. Importantly, members of the *Lactobacillus* genus, highly abundant in our adult macaques (Figure 2), are widely used probiotics with potential effects on lipid metabolism [51]. We also noticed that *Bifidobacterium*, whose members are the key probiotics for the metabolism of oligosaccharides in breast milk [52], also increased with age. The increase of these lactic acid-producing probiotics might indicate a potential role of these bacteria in healthy aging. In addition, *Eubacterium coprostanoligenes* has been identified as a cholesterol-reducing anaerobe [53]. Genera *Christensenellaceae R-7 group*, *Ruminococcaceae UCG–002*, *Ruminococcaceae UCG–010*, and *Lachnospiraceae FCS020 group* were linked to circulating lipid-related metabolites in a recent population-based study [54]. *Candidatus soleaferrea* was increased in a randomized controlled trial of hypocaloric diet with Hass avocado [55]. In line with these findings, changes in microbial functions related to metabolisms of lipids and carbohydrates increased with age (Figure 7). In addition, these microbes positively associated with age have also been implicated in diseases. *Treponema 2*, *Rikenellaceae RC9 gut group*, and *Prevotellaceae UCG–003* were increased in rats with isoproterenol-induced acute myocardial ischemia [56], whereas in a meta-analysis *Christensenellaceae R-7 group* was found to be reduced in patients affected by intestinal diseases [57]. Intriguingly, although the reported role of archaea in the host’s health remained unclear, our results showed that the archaeal family Methanobacteriaceae significantly increased in elderly macaques, and the genus *Methanobrevibacter* increased with age in the macaque gut microbiota. Such findings thus indicate a positive association of these methanogens with host aging.

This study further highlights the pivotal role of driver microbes in age-dependent changes of the gut microbiota (Figures 5 and 6). Genus *Prevotella 9*, with a high abundance in our captive macaques, was identified as the most important hub mediating a large proportion of microbial interactions in the gut microbiota across all ages. And it acted as the key driver responsible for the gut microbiota maturation from infants to young adults. The exact biological significance of *Prevotella 9* in the context of integrative bacterial community and microbiota development has yet to be further elucidated. A recent reanalysis of existing gut metagenomes from NHPs and humans reported that *Prevotella* was prevalent in the primate gut microbiota of different host species [20]. In line with such findings, the *Prevotella 9* genus was highly abundant across all ages with the gradual age-dependent decrease in our captive macaques. The high abundance of *Prevotella* in both humans and NHPs could be strongly associated with plant-based, low-fat diets [24], and implicated host–microbiota coevolution [58]. Nevertheless, the abundance of *Prevotella 9* in adult macaques decreased with age, along with a rise in the abundance of other microbes such as the *Rikenellaceae RC9 gut group* and *Megasphaera*, pointing to age-dependent microbiota development. Such shifts of driver microbes could in turn impact the phenotypes and functions of gut microbiota.

Compared to rodent animals widely used in experiments, NHPs have a much longer life span. Crab-eating macaques usually live longer than 20 years in captivity. Therefore, we used a relatively feasible cross-sectional study design in the current study, although prospective studies could provide more confident evidence in the future. Due to such a long life span, by far, it remains a big challenge to objectively and accurately assess such chronic and long-term age effects in an NHP model through experiments. Moreover, the sample size of NHP model studies is usually limited by animal ethics and the higher costs compared to conventional rodent studies. Our findings thus warrant further studies of the gut microbiota in captive NHPs.

## Conclusion

In summary, by using captive crab-eating macaques to control confounding factors, the current study demonstrates evident age-dependent structural and functional changes in the healthy gut microbiota during the host’s development and aging. Our key findings of age-associated microbes, composed of both commensals and suspicious pathogens, indicate the potential importance of appropriate bacterial exposure for the early development of the host. Moreover, the hub and driver microbes identified by network topology analysis probably play a pivotal role as core microbes in microbial communities, and are responsible for the maturation and development of the primate gut microbiota. By characterizing the age-dependent changes in the healthy gut microbiota, the current study also provides a baseline for comparison and understanding of disease-related changes in the primate gut microbiota.

## Materials and methods

### Animals used in the study

A total of 104 male crab-eating macaques from Guangdong Xiangguan Biotechnology Co. Ltd. (Guangzhou, China) were included in the current study. All the animals were kept in a well-controlled environment with moderate room temperature (16 °C**–**28 °C) and relative humidity of 40%**–**70%, as well as a 12-h light/12-h dark cycle. Individual animals were kept in separate cages, and animals at different ages were never mixed for co-housing. The animals were confirmed to be in good health by records and veterinary examination prior to the study. These animals were fed a normal formula chow diet, and regularly examined by veterinarians. In addition, to ensure no infection, their fur, skin, rectal swab, and blood samples were regularly tested by the government agency and a third-party laboratory to ensure that these animals were negative for specific pathogens (*Salmonella*, *Shigella*, and germatogenic fungi), viruses (simian T-lymphotropic virus 1, simian retrovirus, simian immunodeficiency virus, and simian herpesviruses), and parasites (ectoparasites and *Toxoplasma gondii*) as shown in Figures S10 and S11. To avoid possible effects of pregnancy in females, only male crab-eating monkeys were included in this study.

The 104 male macaques included in the current study were composed of four different age groups, including the infant (1–2 years old, n = 26), young-adult (4–6 years old, n = 26), middle-aged (7–10 years old, n = 26), and elderly (≥ 13 years old, n = 26) groups. For the infant group, post-weaning infant macaques were selected to reduce the possible effects of breastfeeding.

### Stool sample collection and DNA extraction

Rectal swab samples were freshly collected from each monkey, and stored at −80 °C immediately until DNA extraction in August, 2018. Microbial DNA was extracted using the TIANamp Stool DNA Kit (Catalog No. DP328, Tiangen, Beijing, China) according to the manufacturer’s instructions, and its concentration and quality were assessed using a Nanodrop One Microvolume UV Spectrophotometer (ThermoFisher Scientific, Waltham, MA).

### 16S rRNA gene sequencing

The hypervariable V4 regions of bacterial/archaeal 16S rRNA genes were amplified using PCR and V4-specific primers (515F: 5′-GTGCCAGCMGCCGCGGTAA-3′ and 806R: 5′-GGACTACHVGGGTWTCTAAT-3′). PCR products were checked using the 2% agarose gel, purified using the GeneJET Gel Extraction Kit (Catalog No. K0691, ThermoFisher Scientific), and sequenced on an Ion S5XL sequencer (ThermoFisher Scientific) with a single-end 400-bp read length configuration.

### Processing of 16S rRNA gene sequencing data

Bioinformatic analysis of the 16S rRNA gene sequencing data was performed using the QIIME2 (version 2018.6.0) analysis pipeline [59]. Briefly, sequencing data were processed by the dada2 program to filter low-quality and chimeric sequences, and generate unique feature tables equivalent to OTU tables at exact match or 100% sequence similarity. Taxonomy was then assigned to these features using the q2-feature-classifier against the full-length SILVA database (release r132) at a 99% similarity cutoff [60]. Analysis of microbiota diversities was conducted in QIIME2: alpha diversity metrics included Pielou’s evenness, phylogenetic diversity, observed OTUs, Shannon index, and Simpson index, and beta diversity metrics included weighted/unweighted UniFrac distances and Bray-Curtis dissimilarity. Comparison of beta diversity was performed using the nonparametric method PERMANOVA. Abundances of OTUs were compared among groups by using STAMP (version v2.1.3) [61]. The LEfSe algorithm was used with a log_10_ LDA score cutoff of 2 to identify taxa specifically increased or exhibiting the highest abundance in particular age groups compared to other groups [62]. Phylogenetic cladograms of LEfSe results were visualized using the GraPhlAn tool (version 1.1.3; https://bitbucket.org/nsegata/graphlan).

### Microbial interactive network construction and analysis

The SparCC algorithm (https://bitbucket.org/yonatanf/sparcc) [63] and the WGCNA [64] were used to estimate the correlations among gut microbes. 100 bootstrap replicates were used to calculate the pseudo *P* values in the SparCC analysis, and correlations with |correlation coefficient (*r*)| > 0.2 and *P* < 0.01 were considered significant. For each OTU with significant SparCC correlation, a weighted node connectivity score was calculated as an indicator of its weight in the network by summing up its |*r*| with all of its first neighbors [65]. WGCNA was conducted using a merging module threshold of 0.8, at least 8 species per module, and a related network weight of 0.7. The constructed SparCC network was further visualized using Cytoscape (version 3.7.0) [66]. The cytoHubba plugin (version 0.1) was used to identify hub genera in the networks [67]. Two node ranking methods, including a local-based method MCC and a global-based method EC, were used to evaluate the importance of genera. In addition, NetShift (https://web.rniapps.net/netshift/) was used to evaluate potential driver microbes using a case-control strategy to compare a pair of networks as described [68]. Neighbor shift (NESH) scores were calculated to quantify increased interactions in the case over the control.

### Prediction of microbial phenotypes and function profiles

The BugBase (https://bugbase.cs.umn.edu/) analysis tool was utilized to predict high-level phenotypes in fecal microbiome samples. PICRUSt (version 1.1.4) was used to predict microbial functions from the 16S rRNA gene sequencing data, which were further categorized using the BRITE hierarchy of the KEGG database [69]. PCA based on KEGG module abundances was conducted using STAMP.

### Statistical analysis

Statistical analysis was performed using GraphPad Prism (version 7.0a; GraphPad software) and the R statistical language (version 3.6.0). The abundances of OTUs and KEGG modules among groups were compared using the non-parametric Kruskal-Wallis test, and evaluated for pairwise inter-group differences with the Tukey’s post-hoc test if overall significance was found. The Benjamini-Hochberg false discovery rate (FDR) correction was applied for multiple testing. Correlations of OTUs, microbial phenotypes, and KEGG functions with age as continuous variables were determined using Spearman correlation analysis. Differences in the taxa were analyzed by LEfSe with default settings.

## Ethical statement

This study complied with protocols approved by the Animal Ethics Committees of Institute of Zoology, Guangdong Academy of Sciences, China, and followed the Guide for the Care and Use of Laboratory Animals [70].

## Data availability

The raw datasets of 16S rRNA gene sequencing in the current study have been deposited in the Genome Sequence Archive [71] at the National Genomics Data Center, Beijing Institute of Genomics, Chinese Academy of Sciences / China National Center for Bioinformation (BioProject: PRJCA003833), and are publicly accessible at https://ngdc.cncb.ac.cn/gsa.

## CRediT author statement

**Zhi-Yuan Wei:** Methodology, Visualization, Writing - original draft. **Jun-Hua Rao:** Conceptualization, Writing - original draft, Writing - review & editing. **Ming-Tian Tang:** Resources. **Guo-An Zhao:** Formal analysis. **Qi-Chun Li:** Methodology, Visualization. **Li-Ming Wu:** Methodology, Visualization. **Shao-Qiang Liu:** Formal analysis. **Bi-Hai Li:** Methodology. **Bai-Quan Xiao:** Methodology, Conceptualization. **Xing-Yin Liu:** Writing - review & editing. **Jian-Huan Chen:** Conceptualization, Writing - original draft, Writing - review & editing, Supervision. All authors have read and approved the final manuscript.

## Competing interests

The authors declared that they have no competing interests.

## Supplementary material

Supplementary Figure S1 **Sparse curve of observed OTUs and sequencing depth**

Supplementary Figure S2 **Alpha diversity metrics of gut microbiota and their age correlation analysis** The alpha diversity metrics in the four age groups and their correlation with age are shown including Pielou’s evenness (A, B), phylogenetic diversity (C, D), observed OTUs (E, F), Shannon (G, H) and Simpson’s indices (I, J) were calculated using Spearman correlation. IF, infants; YA, young adults; MA, the middle-aged; EL, the elderly. *, P < 0.05; **, P < 0.01; ***, P < 0.001.

Supplementary Figure S3 **Differential gut microbial phyla among age groups** Pairwise P values are calculated using nonparametric Kruskal-Wallis test with Tukey post-hoc test. *, P < 0.05; **, P < 0.01; ***, P < 0.001.

Supplementary Figure S4 **Differential gut microbial genera among age groups** Pairwise P-values are calculated using nonparametric Kruskal-Wallis test with Tukey post-hoc test. *, P < 0.05; **, P < 0.01; ***, P < 0.001.

Supplementary Figure S5 **Spearman correlation between differential gut microbial phyla with age**

Supplementary Figure S6 **Spearman correlation between differential gut microbial genera with age**

Supplementary Figure S7 **Microbial cluster (modules and interaction results from WGCNA** The networks were shown for different age groups (A–D) and all monkeys in the study (E), using with the threshold of module merging equal to 0.8, at least 8 species per module, and the weight of related networks equal to 0.7. WGCNA, weighted correlation network analysis.

Supplementary Figure S8 **Comparison of additional gut microbial phenotypes predicted by BugBase among the four age groups** Pairwise P-values are calculated using nonparametric Kruskal-Wallis test with Tukey post-hoc test. *: P < 0.05; **: P < 0.01; ***: P < 0.001.

Supplementary Figure S9 **Interactive network between age-associated gut microbial genera and PICRUSt-predicted KEGG modules** The network is constructed from the Spearman correlation between age-associated gut microbial genera and KEGG modules. Correlations with |r| > 0.7 and P < 0.05 are shown. Node sizes denote the sum of |r| of the genus from all of its correlations with KEGG modules. Nodes are color green if negatively correlated with age, and yellow if positively correlated with age. Eclipses denote age-associated microbial genera, and diamonds denote age-associated KEGG modules. Genera with their name colored red were driver microbes identified by NetShift analysis. Edges are colored red if r >0, and blue if r < 0. The thicker the line and the darker the color mean the absolute value of the correlation coefficient.

Supplementary Figure S10 **An example of sample test report from the administrative agency** The original report is in Chinese. Sample from macaque fur, skin, rectal swab, and blood are tested to ensure no infection by specific pathogens (Salmonella, Shigella, and dermatogenic fungi), and parasites (ectoparasites and Toxoplasma gondii).

Supplementary Figure S11 **An example of sample test report from a third-party testing laboratory** The report is in Chinese. Macaque serum samples are tested for detection of viruses (simian T-lymphotropic virus 1, simian retrovirus, simian immunodeficiency virus, and simian herpesviruses). All of the NHP animals in the current study are tested negative.
