# Supplementary figures and images for: Characterization of Changes and Driver Microbes in Gut Microbiota During Healthy Aging Using A Captive Monkey Model
Source: Genomics Proteomics Bioinformatics. 2021 Dec 30;20(2):350–65. doi: 10.1016/j.gpb.2021.09.009 (PMC9684162; doi:10.1016/j.gpb.2021.09.009)

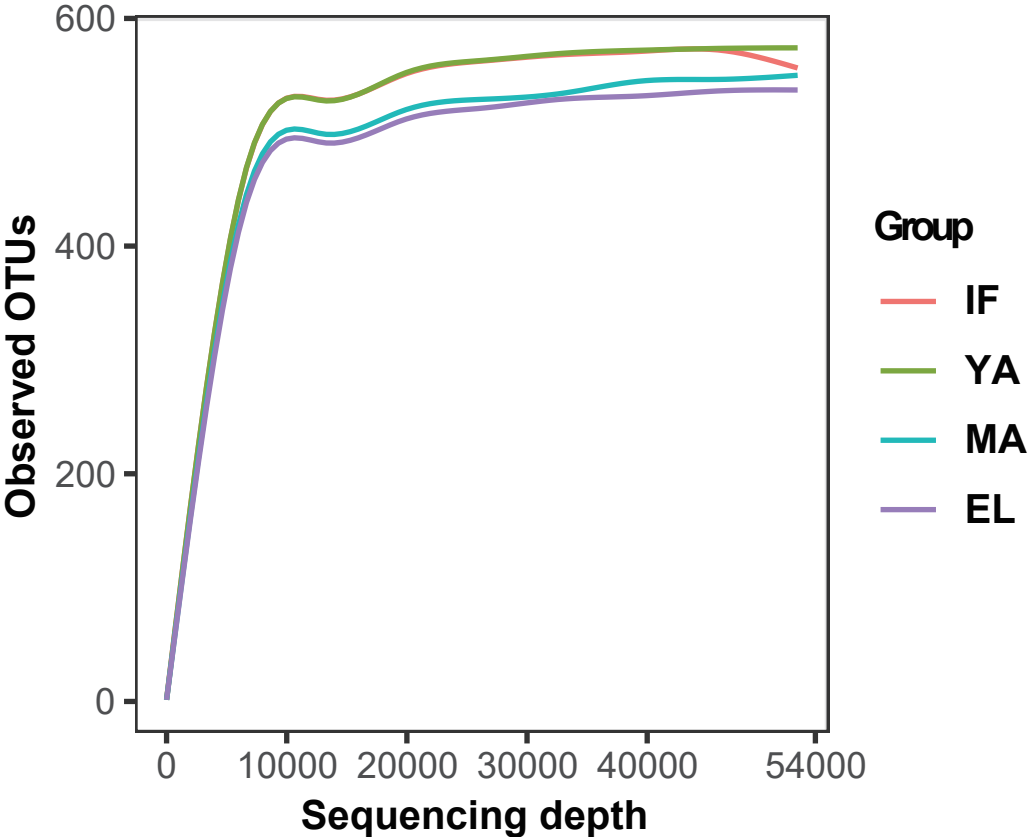

Supplement: Supplementary Figure S1 — Sparse curve of observed OTUs and sequencing depth [file mmc1.pdf]

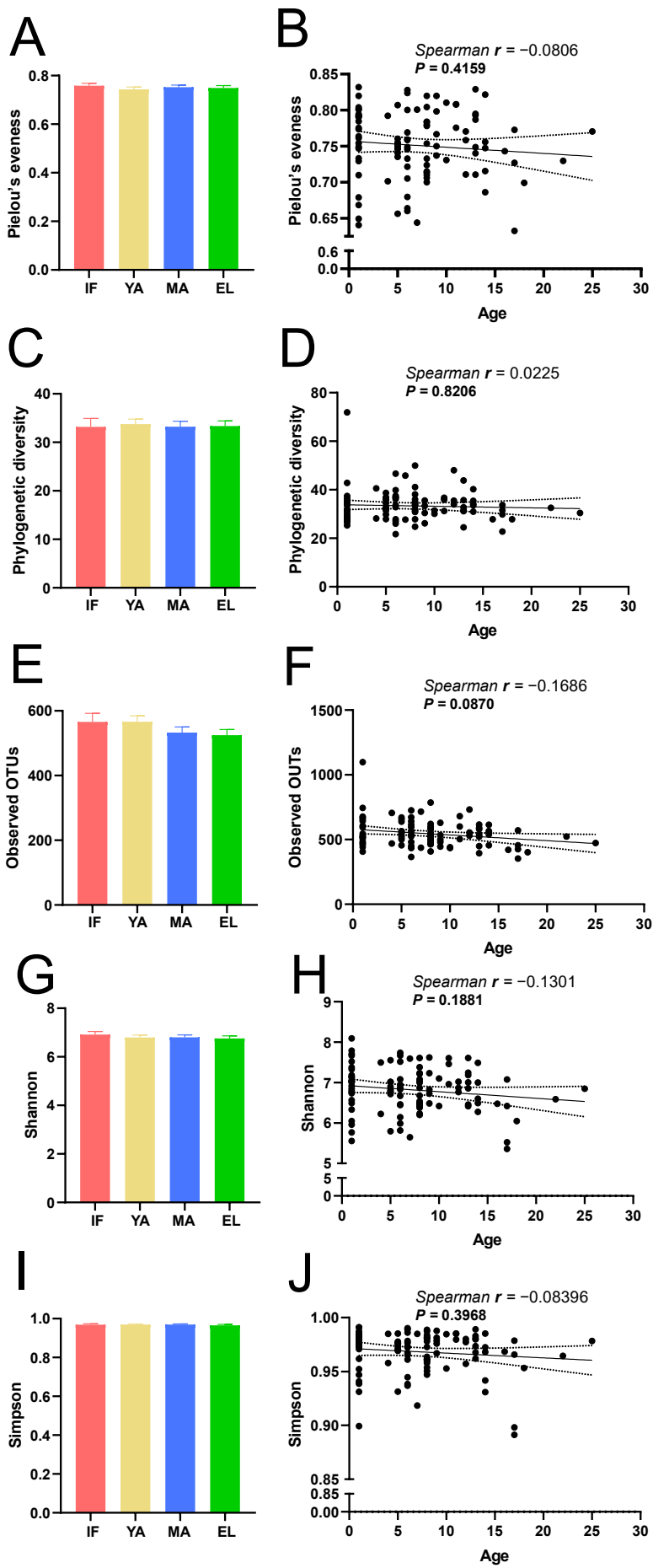

Supplement: Supplementary Figure S2 — Alpha diversity metrics of gut microbiota and their age correlation analysis The alpha diversity metrics in the four age groups and their correlation with age are shown including Pielou’s evenness (A, B), phylogenetic diversity (C, D), observed OTUs (E, F), Shannon (G, H) and Simpson’s indices (I, J) were calculated using Spearman correlation. IF, infants; YA, young adults; MA, the middle-aged; EL, the elderly. *, P < 0.05; **, P < 0.01; ***, P < 0.001. [file mmc2.pdf]

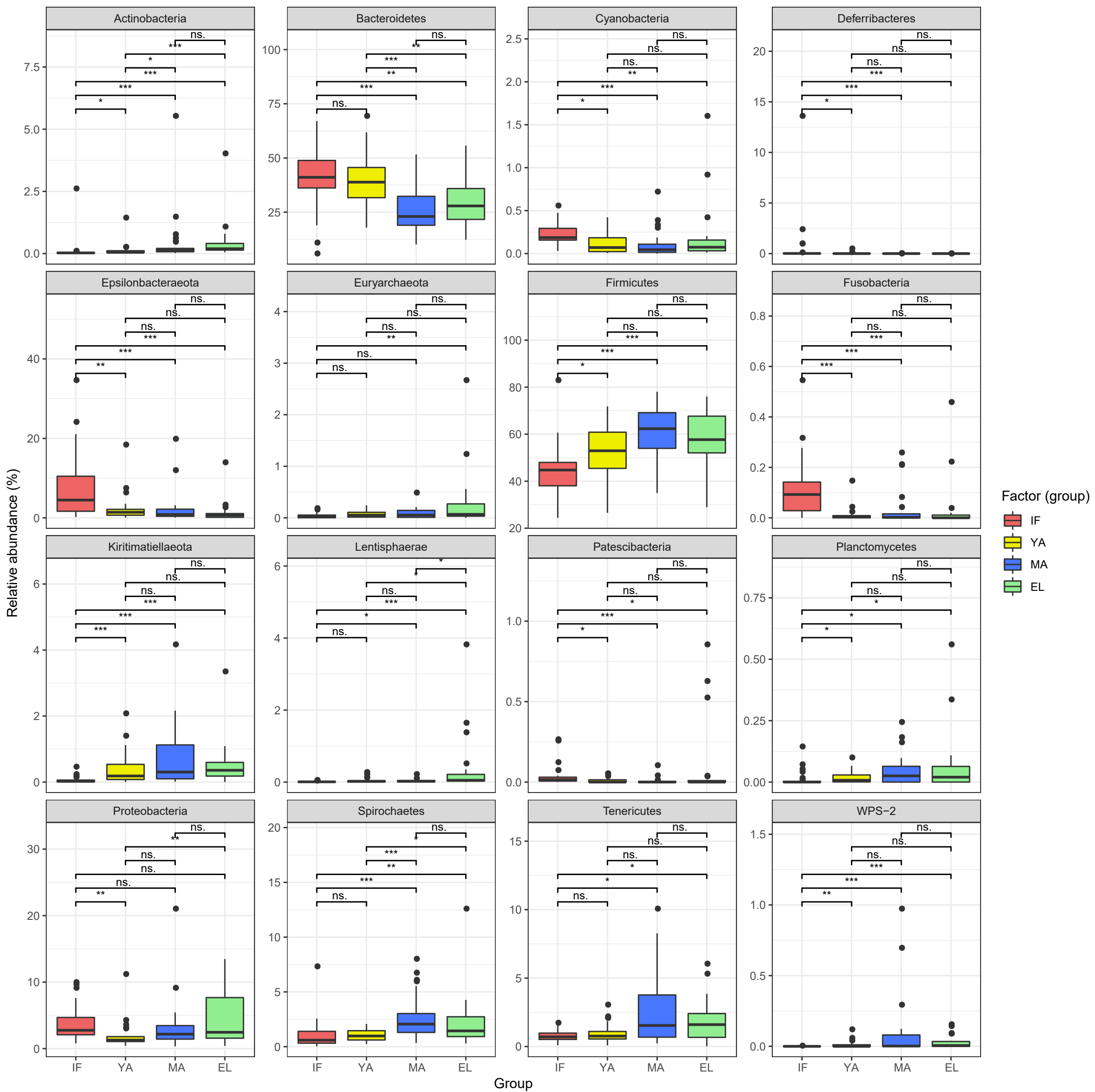

Supplement: Supplementary Figure S3 — Differential gut microbial phyla among age groups Pairwise P values are calculated using nonparametric Kruskal-Wallis test with Tukey post-hoc test. *, P < 0.05; **, P < 0.01; ***, P < 0.001. [file mmc3.pdf]

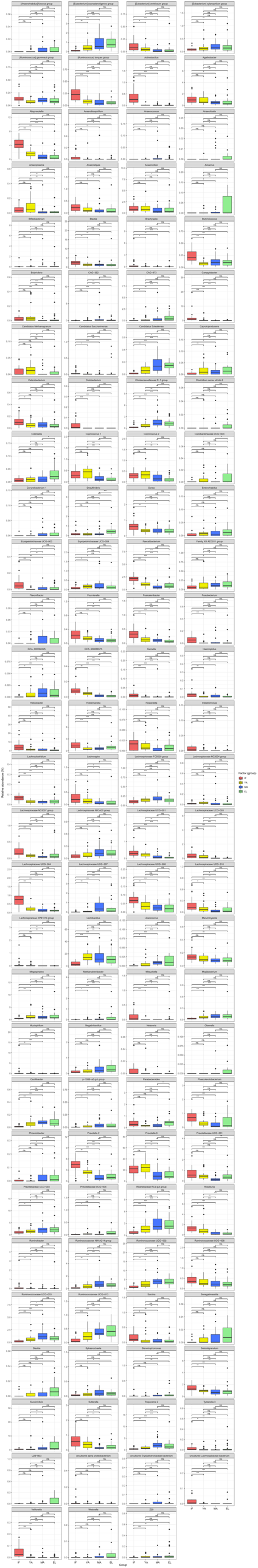

Supplement: Supplementary Figure S4 — Differential gut microbial genera among age groups Pairwise P-values are calculated using nonparametric Kruskal-Wallis test with Tukey post-hoc test. *, P < 0.05; **, P < 0.01; ***, P < 0.001. [file mmc4.pdf]

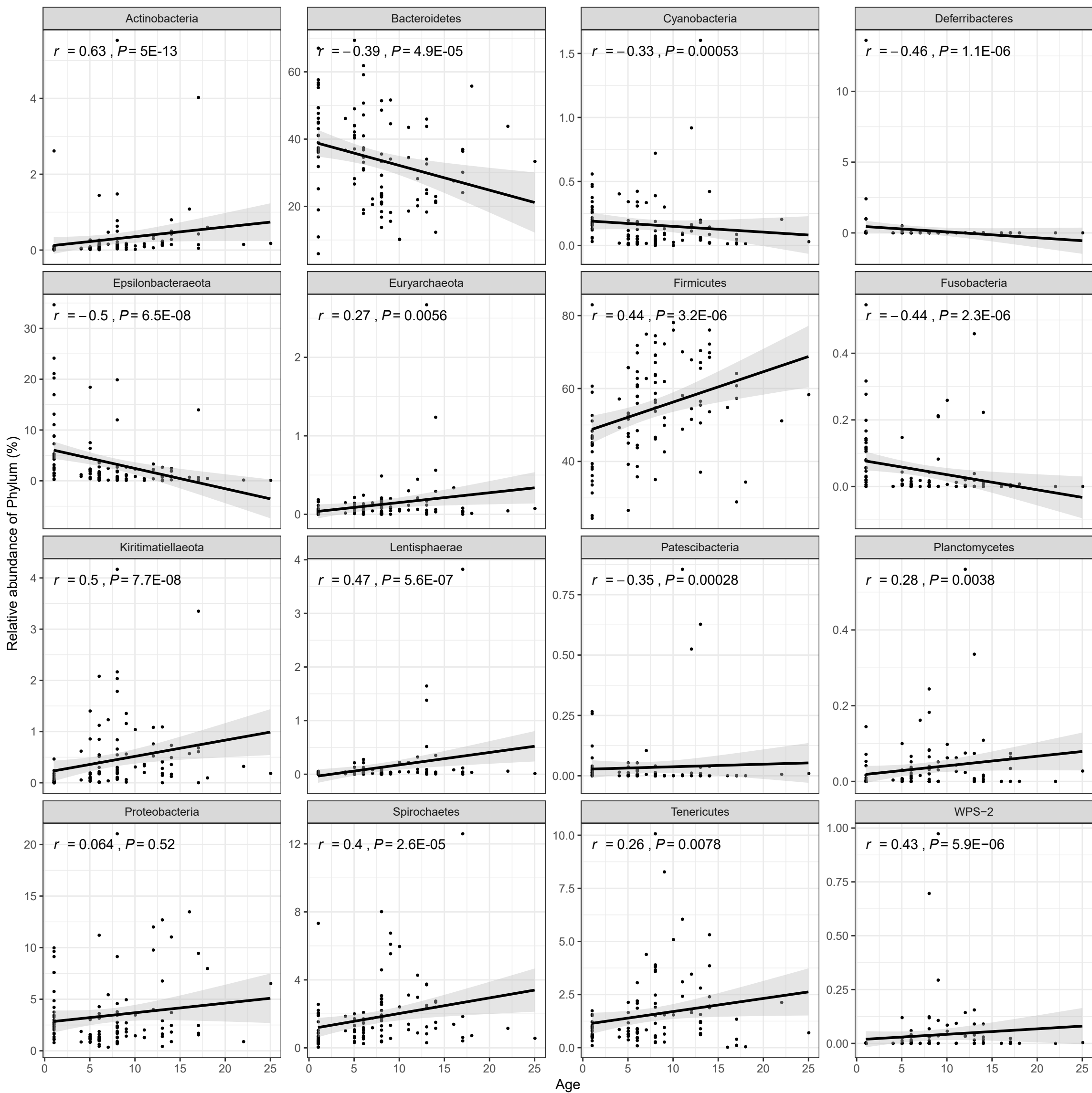

Supplement: Supplementary Figure S5 — Spearman correlation between differential gut microbial phyla with age [file mmc5.pdf]

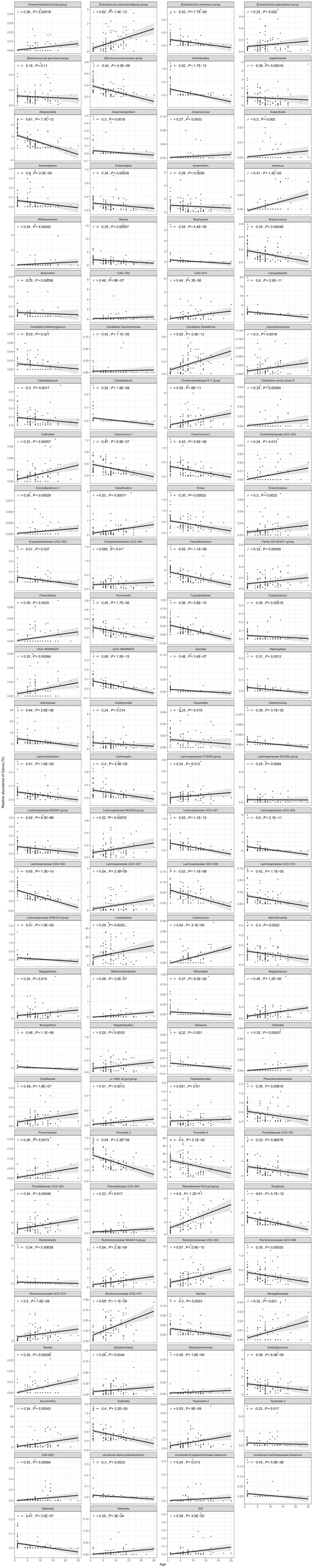

Supplement: Supplementary Figure S6 — Spearman correlation between differential gut microbial genera with age [file mmc6.pdf]

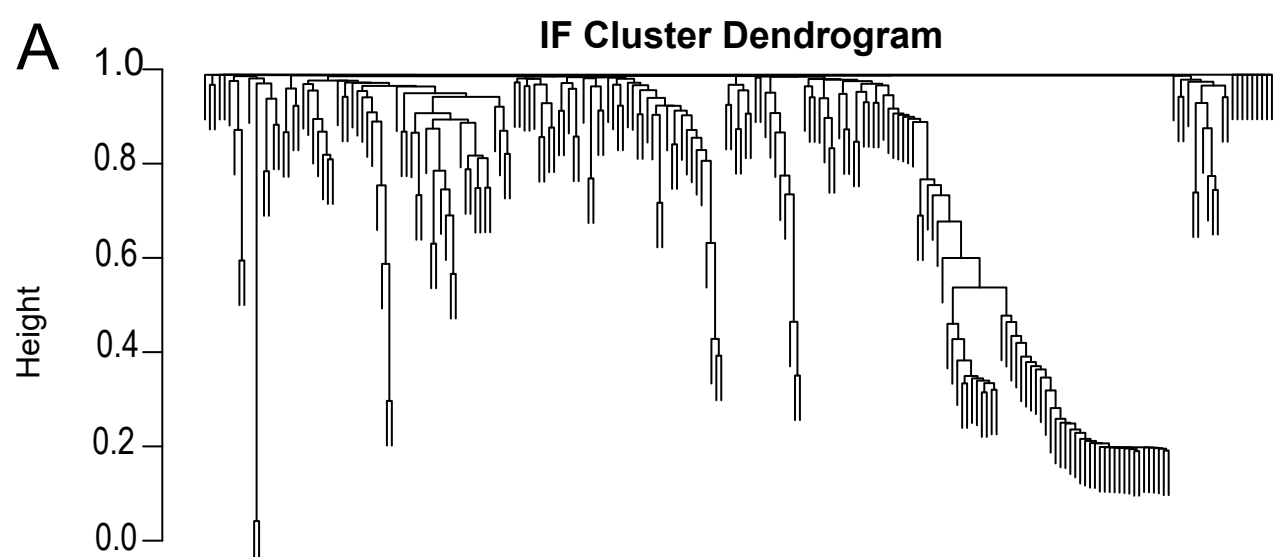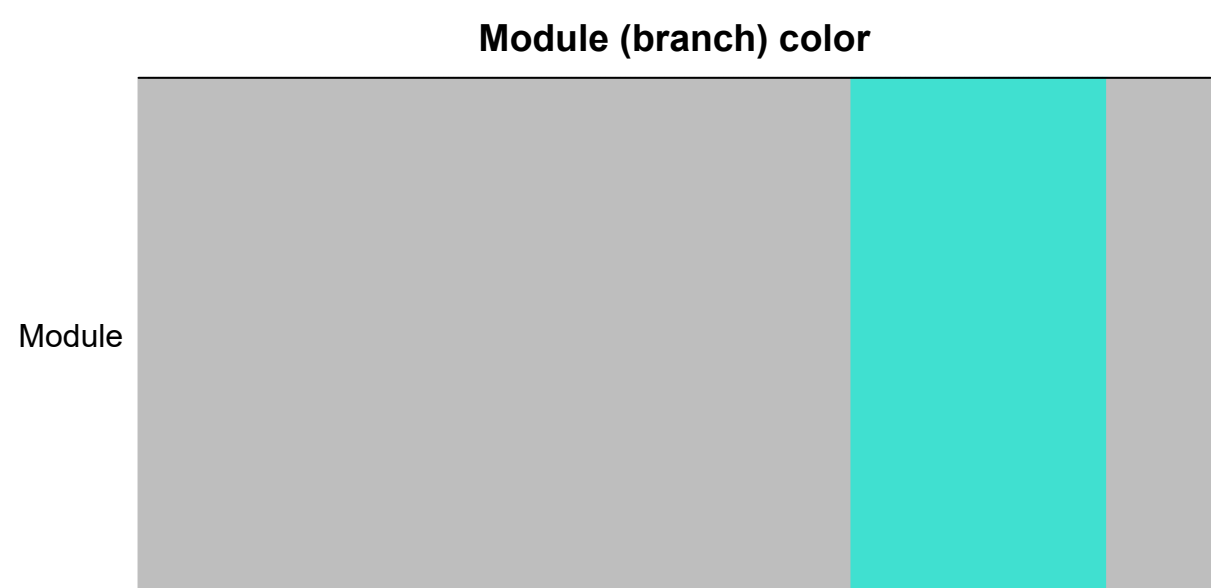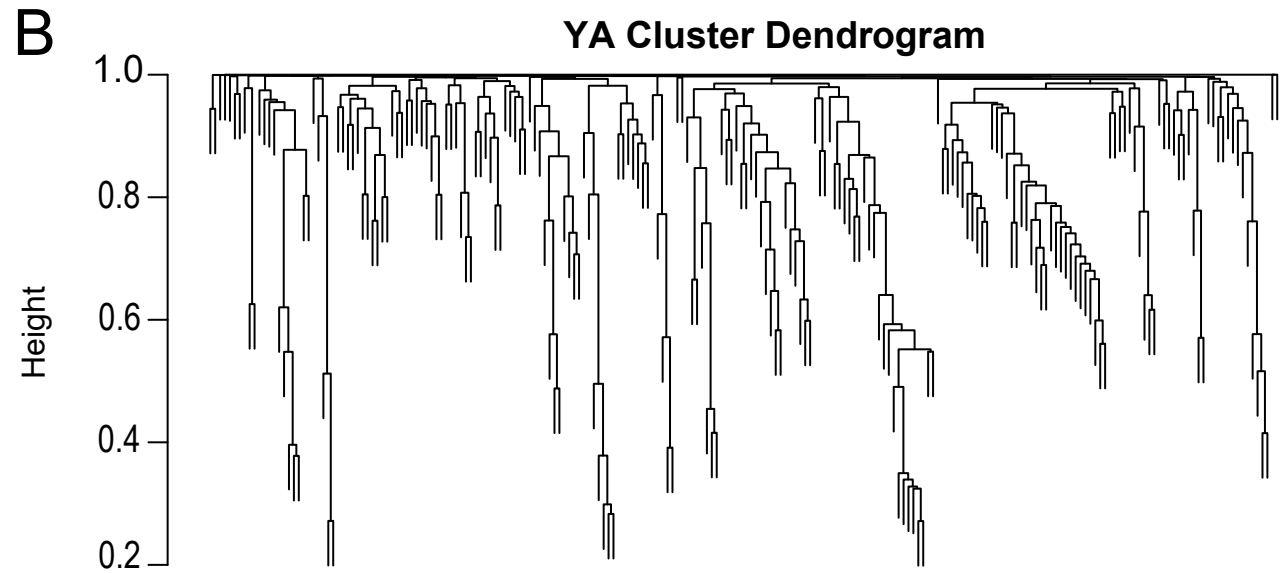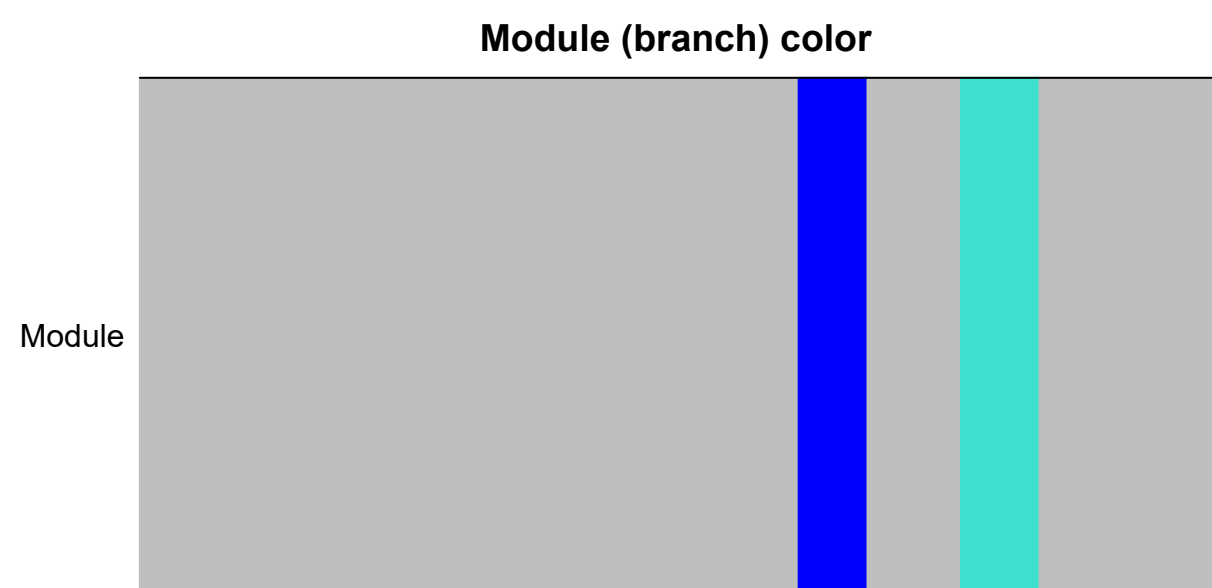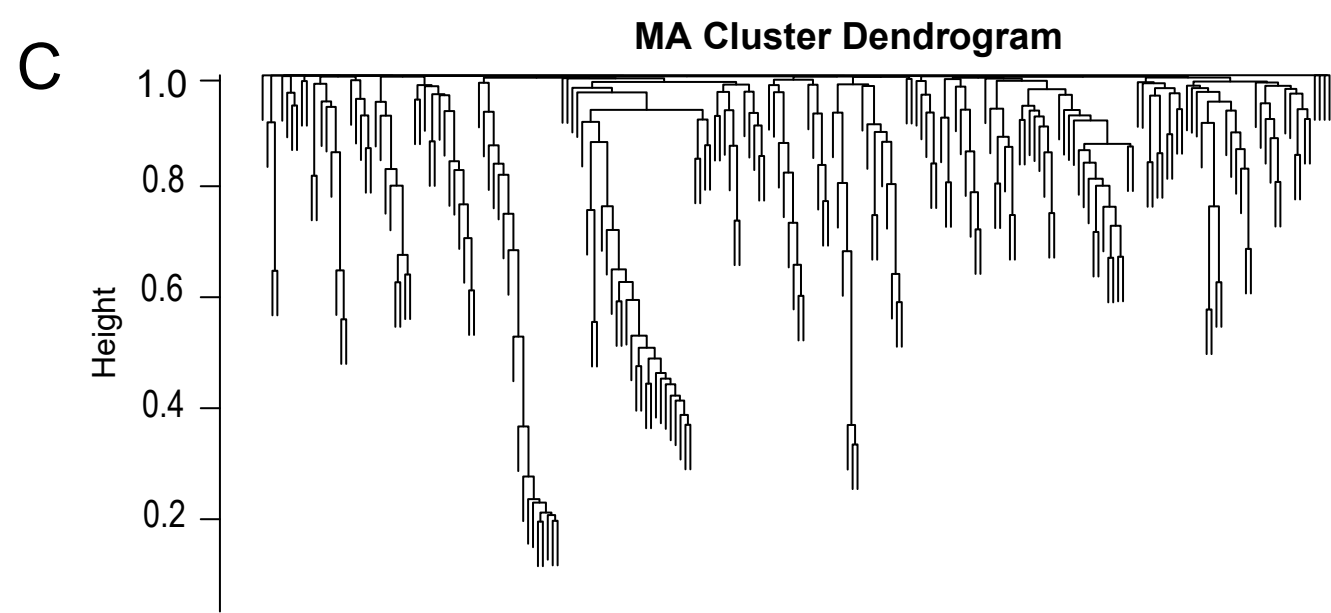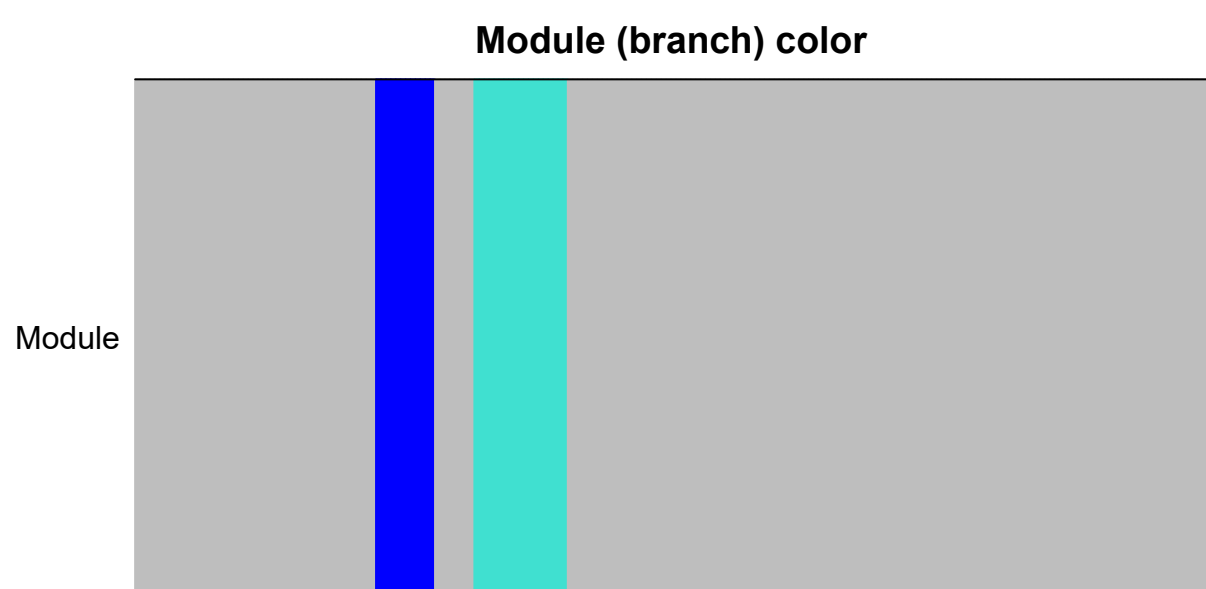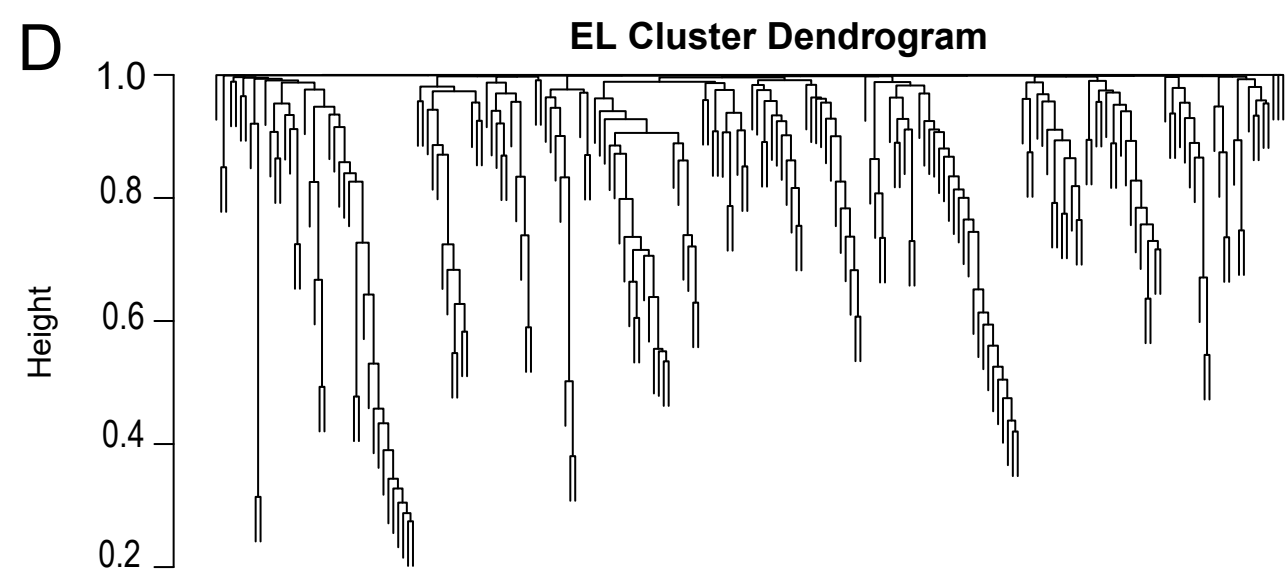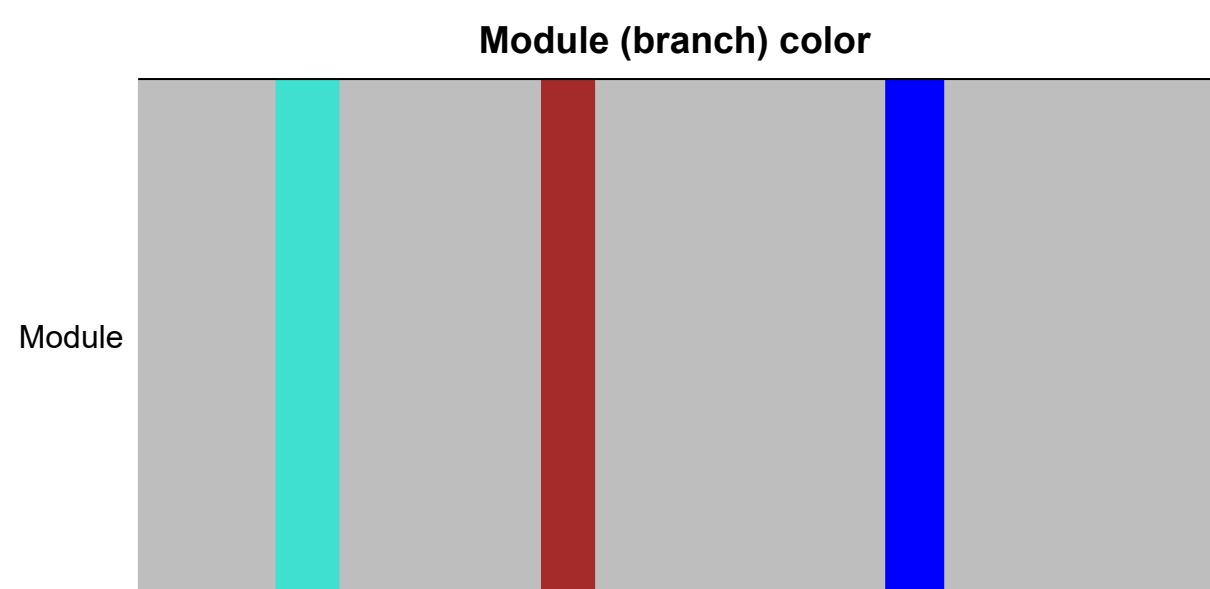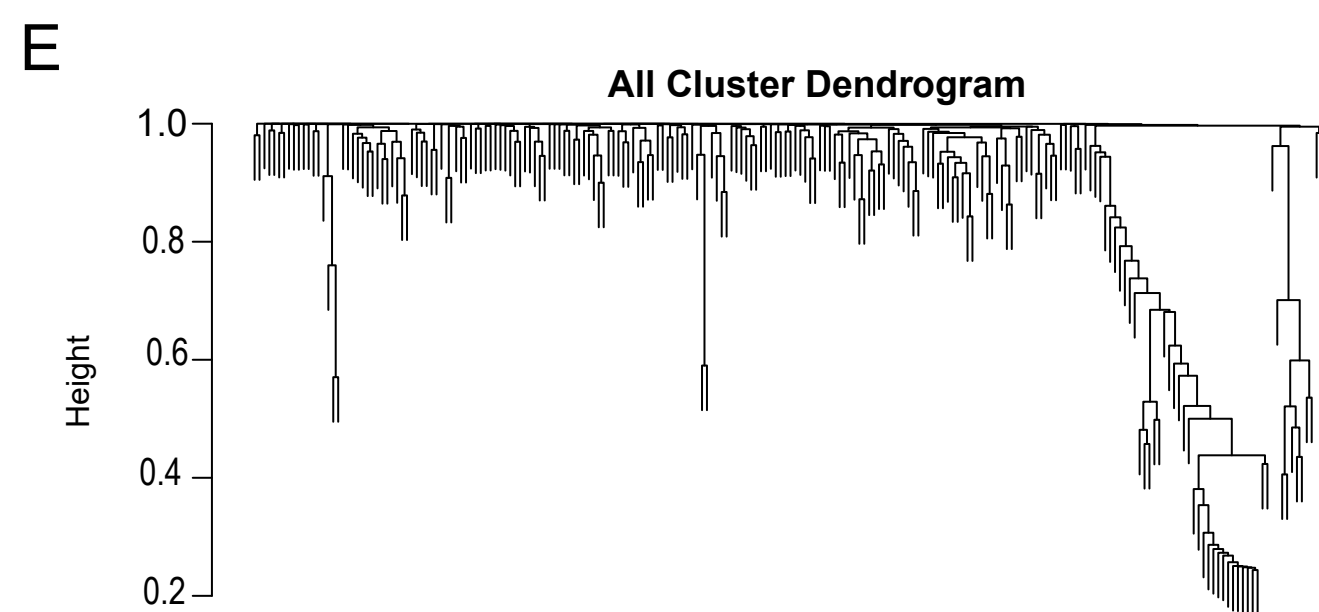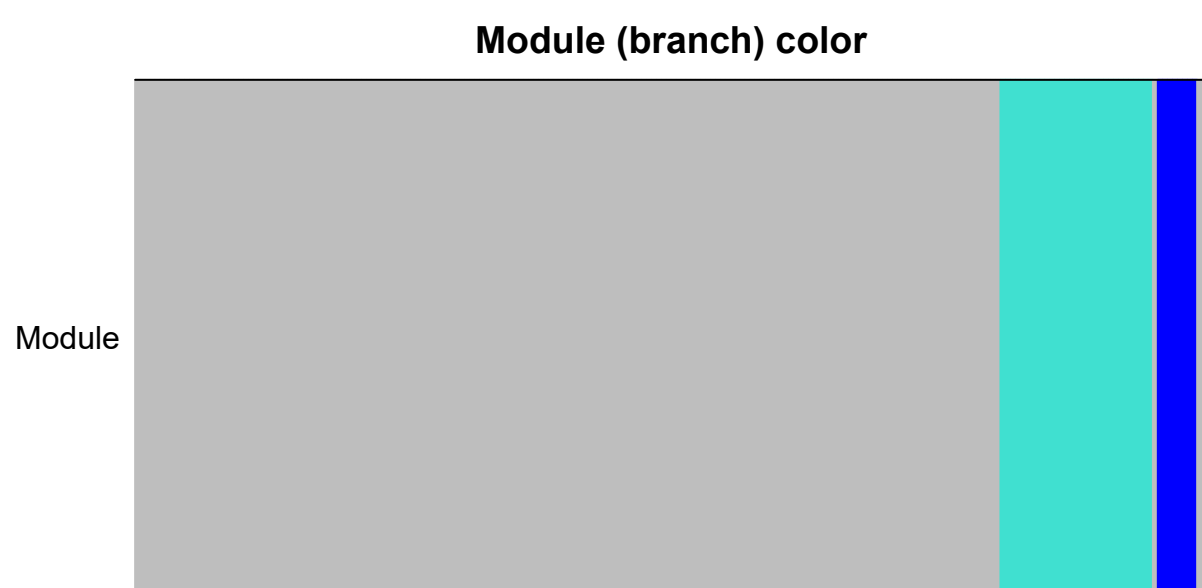

Supplement: Supplementary Figure S7 — Microbial cluster (modules and interaction results from WGCNA The networks were shown for different age groups (A–D) and all monkeys in the study (E), using with the threshold of module merging equal to 0.8, at least 8 species per module, and the weight of related networks equal to 0.7. WGCNA, weighted correlation network analysis. [file mmc7.pdf]

**A**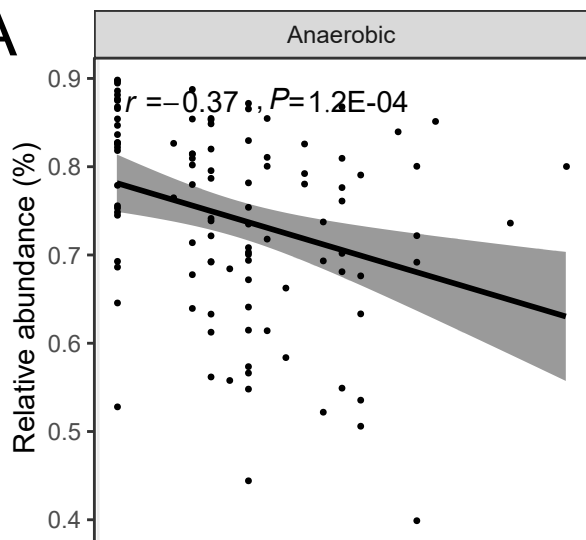**B**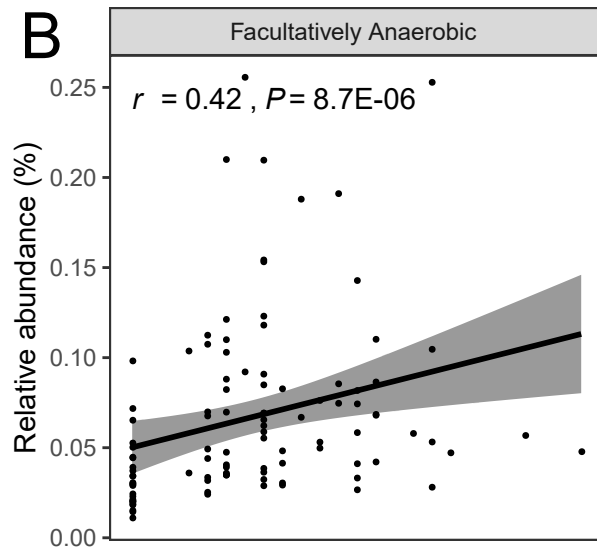**C**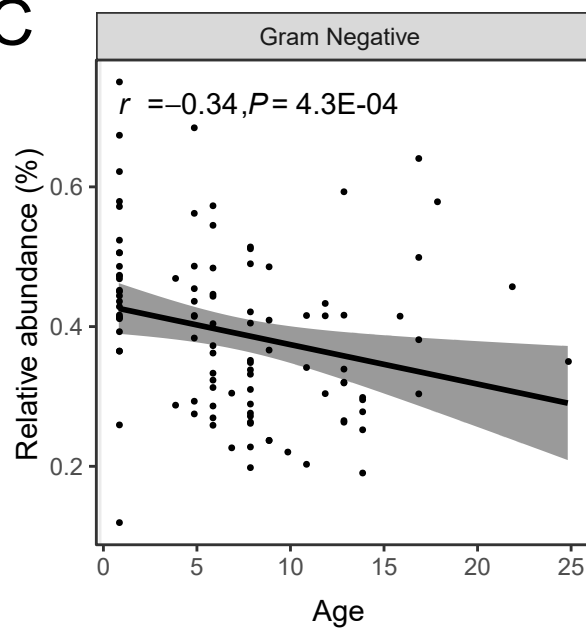**D**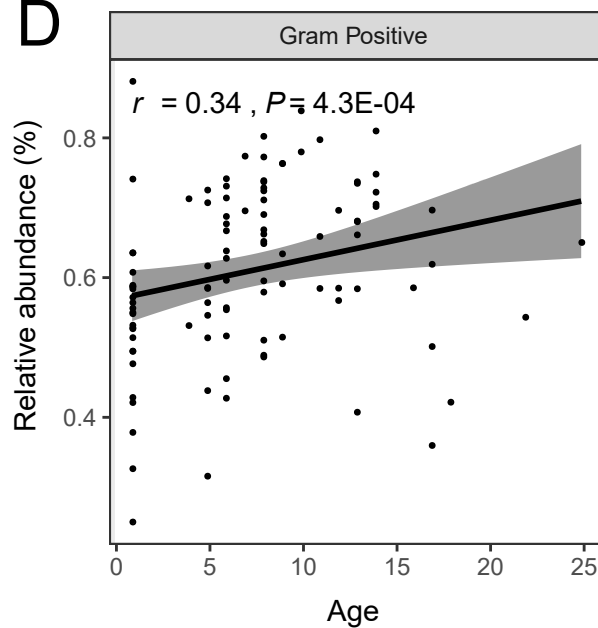

Supplement: Supplementary Figure S8 — Comparison of additional gut microbial phenotypes predicted by BugBase among the four age groups Pairwise P-values are calculated using nonparametric Kruskal-Wallis test with Tukey post-hoc test. *: P < 0.05; **: P < 0.01; ***: P < 0.001. [file mmc8.pdf]

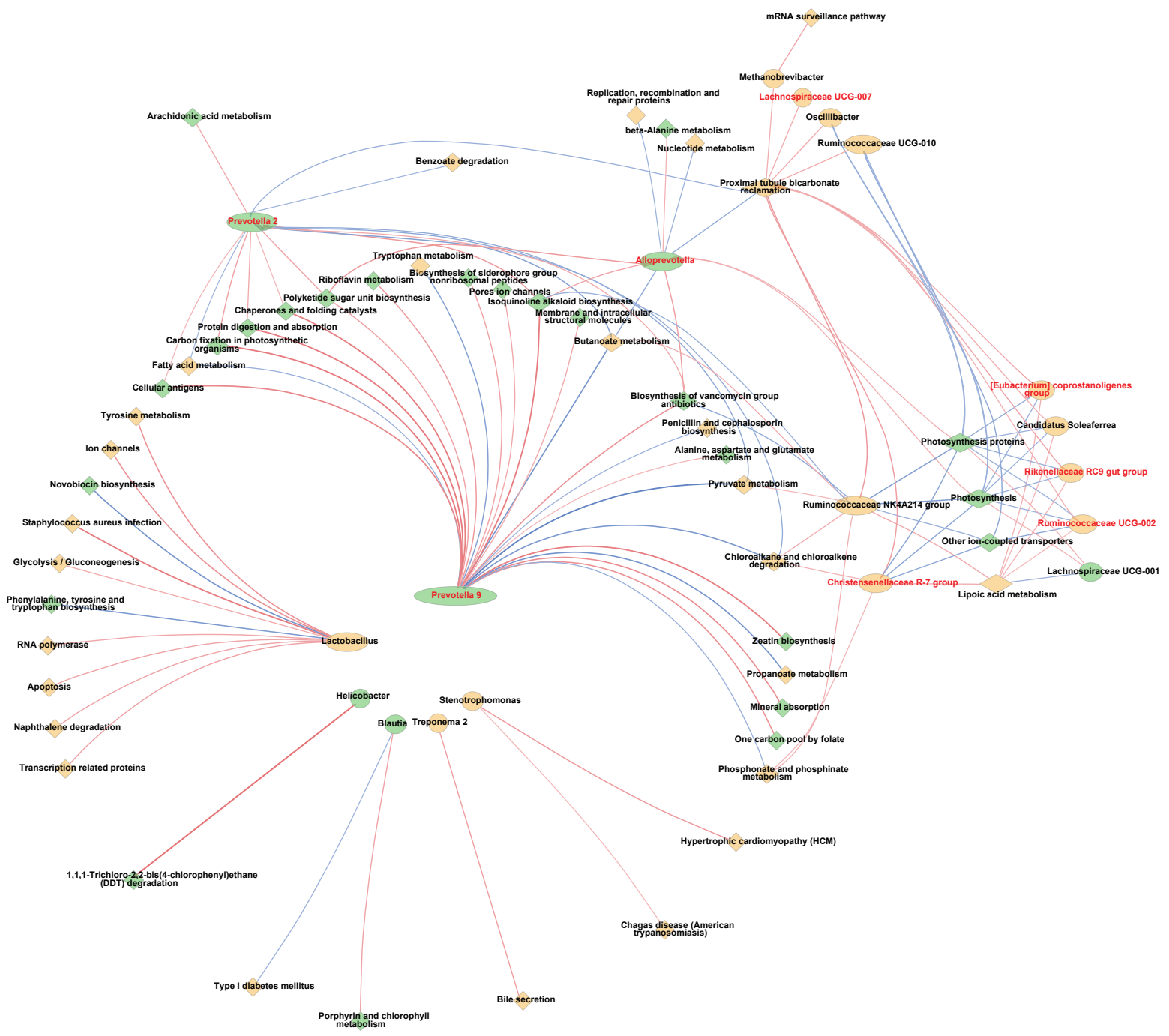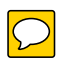

Supplement: Supplementary Figure S9 — Interactive network between age-associated gut microbial genera and PICRUSt-predicted KEGG modules The network is constructed from the Spearman correlation between age-associated gut microbial genera and KEGG modules. Correlations with |r| > 0.7 and P < 0.05 are shown. Node sizes denote the sum of |r| of the genus from all of its correlations with KEGG modules. Nodes are color green if negatively correlated with age, and yellow if positively correlated with age. Eclipses denote age-associated microbial genera, and diamonds denote age-associated KEGG modules. Genera with their name colored red were driver microbes identified by NetShift analysis. Edges are colored red if r >0, and blue if r < 0. The thicker the line and the darker the color mean the absolute value of the correlation coefficient. [file mmc9.pdf]
