# Supplementary material for: Characterization of Changes and Driver Microbes in Gut Microbiota During Healthy Aging Using A Captive Monkey Model
Source: Genomics Proteomics Bioinformatics. 2021 Dec 30;20(2):350–65. doi: 10.1016/j.gpb.2021.09.009 (PMC9684162; doi:10.1016/j.gpb.2021.09.009)
Supplement: Supplementary Figure S10 — An example of sample test report from the administrative agency The original report is in Chinese. Sample from macaque fur, skin, rectal swab, and blood are tested to ensure no infection by specific pathogens (Salmonella, Shigella, and dermatogenic fungi), and parasites (ectoparasites and Toxoplasma gondii). [file mmc10.pdf]

报告编号：No. A2193481
报告共 5 页

2016191225Z
2019(粤)质监认字294号

中国认可
国际互认
检测
TESTING
CNAS L3276

检 验 报 告

受检单位：广州相观生物科技有限公司

样品名称：猴

检验级别：普通级

检验类别：监督

广东省实验动物监测所
广东省质量监督实验动物检验站

广东省实验动物监测所
广东省质量监督实验动物检验站
检 验 报 告

样品名称	猴	品种/品系	食蟹猴
等级	普通级	抽(送)样表号	2019643
受检单位	广州相观生物科技有限公司	检验类别	监督
委托单位	广东省科学技术厅	样品数量	20只
抽样地点	广州市从化鳌头镇横坑村菱角队白编104号首层6栋	抽样基数	4000只
来样方式	抽样	抽(送)样日期	2019-12-04
样品编号	A2019348	样品状态描述	皮毛、皮屑、肛拭子、血液
样本编号	A2019348-1～A2019348-20		
检验依据	GB 14922.1-2001 《实验动物 寄生虫学等级及监测》、GB 14922.2-2011 《实验动物 微生物学等级及监测》		
检验结论	本次检验结果符合GB 14922.1-2001 《实验动物 寄生虫学等级及监测》、GB 14922.2-2011 《实验动物 微生物学等级及监测》标准中普通级动物质量的指标要求，检验合格。<br>（广东省质量监督实验动物检验站 检验专用章）		
备注	/		

编制人：刘佳乐　　验核人：[illegible]　　签发人：张[illegible]

日 期：2019年12月17日　　日 期：2019年12月17日　　日 期：2019年12月23日

广东省实验动物监测所
广东省质量监督实验动物检验站
检　验　报　告

样本编号	A2019348-1	A2019348-2	A2019348-3	A2019348-4	A2019348-5
对应受检单位自编号	/	/	/	/	/
样本状态描述	皮毛、皮屑、肛拭子、血液	皮毛、皮屑、肛拭子、血液	皮毛、皮屑、肛拭子、血液	皮毛、皮屑、肛拭子、血液	皮毛、皮屑、肛拭子、血液
样本编号	A2019348-6	A2019348-7	A2019348-8	A2019348-9	A2019348-10
对应受检单位自编号	/	/	/	/	/
样本状态描述	皮毛、皮屑、肛拭子、血液	皮毛、皮屑、肛拭子、血液	皮毛、皮屑、肛拭子、血液	皮毛、皮屑、肛拭子、血液	皮毛、皮屑、肛拭子、血液
样本编号	A2019348-11	A2019348-12	A2019348-13	A2019348-14	A2019348-15
对应受检单位自编号	/	/	/	/	/
样本状态描述	皮毛、皮屑、肛拭子、血液	皮毛、皮屑、肛拭子、血液	皮毛、皮屑、肛拭子、血液	皮毛、皮屑、肛拭子、血液	皮毛、皮屑、肛拭子、血液
样本编号	A2019348-16	A2019348-17	A2019348-18	A2019348-19	A2019348-20
对应受检单位自编号	/	/	/	/	/
样本状态描述	皮毛、皮屑、肛拭子、血液	皮毛、皮屑、肛拭子、血液	皮毛、皮屑、肛拭子、血液	皮毛、皮屑、肛拭子、血液	皮毛、皮屑、肛拭子、血液

广东省实验动物监测所
广东省质量监督实验动物检验站
病原菌检验报告单

样品编号： A2019348 受检样品： 皮屑,肛拭子

检验项目	检验方法	标准要求	检验结果	评价
沙门菌	GB/T 14926.1-2001	排除	阴性	合格
皮肤病原真菌	GB/T14926.4-2001	排除	阴性	合格
志贺菌	GB/T 14926.47-2008	排除	阴性	合格

检测日期： 2019 年 12 月 04 日 至 2019 年 12 月 18 日

广 东 省 实 验 动 物 监 测 所

广东省质量监督实验动物检验站
病 毒 检 验 报 告 单

样品编号： A2019348　　受检样品： 血清

检验项目	检验方法	标准要求	检验结果	评价
猕猴疱疹病毒1型（BV）	GB/T 14926.60-2001（ELISA）	排除	抗体阴性	合格

检测日期： 2019 年 12 月 04 日　至 2019 年 12 月 06 日

广 东 省 实 验 动 物 监 测 所

广东省质量监督实验动物检验站
寄 生 虫 检 验 报 告 单

样品编号：A2019348　　受检样品：毛皮,血清

检验项目	检验方法	标准要求	检验结果	评价
体外寄生虫	GB/T 18448.1-2001	排除	阴性	合格
弓形虫（抗体）（IHA）	GB/T 18448.2-2008	排除	阴性	合格

检测日期： 2019 年 12 月 04 日　至　2019 年 12 月 06 日
