# Supplementary material for: Characterization of Changes and Driver Microbes in Gut Microbiota During Healthy Aging Using A Captive Monkey Model
Source: Genomics Proteomics Bioinformatics. 2021 Dec 30;20(2):350–65. doi: 10.1016/j.gpb.2021.09.009 (PMC9684162; doi:10.1016/j.gpb.2021.09.009)
Supplement: Supplementary Figure S11 — An example of sample test report from a third-party testing laboratory The report is in Chinese. Macaque serum samples are tested for detection of viruses (simian T-lymphotropic virus 1, simian retrovirus, simian immunodeficiency virus, and simian herpesviruses). All of the NHP animals in the current study are tested negative. [file mmc11.pdf]

检 测 报 告

TEST REPORT

报告编号：<br>Report No.	RA0806-201841
签发日期：<br>Date of Issue	2020-8-25
送样单位：<br>Submitted By	广州相观生物科技有限公司<br>Guangzhou Xiangguan Biotechnology Co.,Ltd.
委托单位：<br>Requested By	广州相观生物科技有限公司<br>Guangzhou Xiangguan Biotechnology Co.,Ltd.
委托地址：<br>Address	广州市从化区明珠工业园<br>Pearl Industry Park, Conghua District, Guangzhou

苏州西山生物技术有限公司

SUZHOU XISHAN BIOTECHNOLOGY INC. (VRL-ASIA)

声 明

STATEMENT

1. 本检测全部在本公司实验室完成。
All the tests were performed in this laboratory.

2. 本报告仅对客户所送检样本有效，不能完全反映动物健康状况。
Test results shown in this report only reflect the status of the specimens at the time received by our lab, and not guarantee or imply the health status of the animals.

3. 本检测报告一式两份，未加盖本公司印章和批准人签名则无效。
Each testing report is in duplicate. It is invalid without an official stamp and authorized signatures.

4. 本报告由 VRL 实验室系统形成，其真伪性可致电苏州西山生物技术有限公司(VRL-Asia)查询。
Our reports were generated by VRL lab information system. The Authenticity of the report can be verified by phonecall or e-mail to VRL Asia.

5. 对此检测报告有任何争议，请在 30 天内与我们联系。
If you have any dispute to this testing report, please contact us within 30 days.

6. 未经本公司书面许可，委托方不得部分复制本报告、不得擅自使用此检测结果进行不当宣传。
Without our written permission, This report cannot be partly copied, and the test requesting facilities should not use our testing results for improper propaganda.

7. 若此报告被误发到贵处，请及时致电 0512 6298 5955 或发邮件到 vrlchina@vrl.net 通知我们。
If you have received this report by mistake, please call us at: 0512 6298 5955(China) or E-mail us at vrlchina@vrl.net.

8. 本检测报告中所涉及的检品名称、种属/品系、动物等级、样本号的信息均为委托方提供，本公司不负责调查确认这些信息的真实性。
The information of samples in this test report, including "Specimen Name", "Species/Strain", "Animal Grade" and "Specimen I.D.", was provided by the test requesting facilities. Our company is not responsible to investigate or confirm the authenticity of these information."

9. 联络方式：苏州工业园区东长路 18 号中节能产业园 35 幢 215123
18 Dongchang Road, Building 35, SIP, Suzhou city, China
Tel: 0512-6298 6955 / 6298 1611 Fax: 0512-6298 5677.

检 测 报 告

SUMMARY

样 品 信 息：<br>Specimen Info	血清<br>Serum	接 样 日 期：<br>Date Received	2020-8-19
动 物 信 息：<br>Animal Info	食蟹猴<br>Cynomolgus monkey (Macaca fascicularis)	检 测 日 期：<br>Date Tested	2020-8-19~2020-8-25
样 本 状 态：<br>Specimen Status	浅黄色澄清液体<br>Red Clear Liquid		

No.	检测项目<br>TEST ITEM		检测依据<br>TEST BASIS	检测数量<br>TESTED	结果 RESULTS<br>POS(+)	IND(±)	NEG(-)	PEN(?)
1	猴T细胞趋向性病毒I型抗体(STLV-1 Ab)	DIA	01-02-0180	99	0	0	99	0
2	猴逆转录D型病毒抗体(SRV Ab)	DIA	01-02-0180	99	0	0	99	0
3	猴免疫缺陷病毒抗体(SIV Ab)	DIA	01-02-0180	99	0	0	99	0
4	猴B病毒抗体(BV Ab)	DIA	01-02-0180	99	0	0	99	0

主 检 Apprasier: 李静文　审 核 Checker: 徐蓉　批 准 Approver: 王晨娟　日 期 Date: 2020-08-25
